# Storage and Retrieval of Electronic Spin Coherence Using Nuclear Spins in a Vanadyl Paddlewheel

**DOI:** 10.1021/jacs.6c10524

**Published:** 2026-08-10

**Authors:** Matteo Lanza, Olga Mironova, Claudio Bonizzoni, Giacomo Bellini, Alessio Nicolini, Alberto Ghirri, Rodolphe Clérac, Mathieu Rouzières, Andrea Cornia, Marco Affronte

**Affiliations:** † Dipartimento di Scienze Fisiche, Informatiche e Matematiche, 9306 Università degli Studi di Modena e Reggio Emilia, via G. Campi 213/A, 41125 Modena, Italy; ‡ Istituto Nanoscienze—CNR, S3, via G. Campi 213/A, 41125 Modena, Italy; § Dipartimento di Scienze Chimiche e Geologiche e UdR INSTM, Università degli Studi di Modena e Reggio Emilia, via G. Campi 103, 41125 Modena, Italy; ∥ Univ. Bordeaux, CNRS, CRPP, UMR 5031, F-33600 Pessac, France

## Abstract

Electronic and nuclear spins are complementary carriers of quantum information: whereas nuclear spins exhibit long coherence times suitable for information storage, electronic spins ensure rapid addressing and readout. A key challenge is harvesting the benefits of both spin degrees of freedom by transferring coherence between them. In this work, we achieved this goal by working on a molecular architecture that affords precise control over the hyperfine multiplets, spin dispersion, and molecular orientation within a crystalline host. As a model system, we used heterobimetallic paddlewheel complex [VOPt­(SOCPh)_4_] diluted in [TiOPt­(SOCPh)_4_]·2THF diamagnetic crystalline host matrix (nominal concentrations of 1 and 10%). In [VOPt­(SOCPh)_4_], vanadyl electronic spin *S* = 1/2 is hyperfinely coupled to the nuclear spin *I* = 7/2 of ^51^V. These electronic and nuclear spins were simultaneously manipulated using sequences of radiofrequency (RF) and microwave (MW) pulses generated by a multifrequency on-chip setup operating at a cryogenic temperature. First, Electron-Nuclear DOuble Resonance (ENDOR) experiments were performed to show the readout of the ^51^V nuclear spin transitions through the electronic spin echo. Further experiments with a dedicated RF-MW pulse sequence demonstrated that electronic phase coherence can be stored in the nuclear spins and retrieved (Storage and Retrieval, SR) after a total free precession time that greatly exceeds the electronic phase memory time. This result demonstrates the possibility of simultaneous manipulation of electronic and nuclear spins and establishes a route for encoding quantum information on both degrees of freedom of a chemically tunable platform.

## Graphical abstract

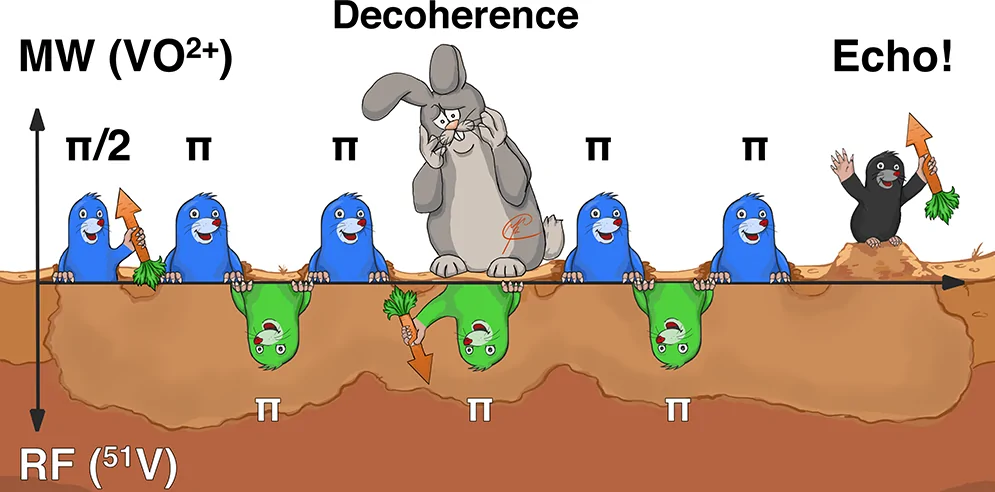

## 1 Introduction

A key challenge in quantum science and technology (QST) is expanding the Hilbert space to a qudit (a higher-dimensional version of a qubit), thereby enhancing the capabilities of quantum information processing (QIP). This necessarily goes along with the development of multilevel (and multifrequency) protocols, and has established, for instance, superconducting devices like fluxonium^1^ as one of the most advanced technologies for quantum computing^2^ and sensing.^3,4^ Another natural choice for implementing and testing multilevel approaches is given by electronic and nuclear spin degrees of freedom. Owing to their large magnetic moments, electronic spins can be addressed by microwave (MW) radiation, enabling fast manipulation at relatively low power (from μW up to mW in 3D cavities, and down to pW using state-of-the-art MW superconducting resonators).^5^ However, their strong interaction with the environment results in shortened coherence times, typically ranging from fractions to hundreds of μs.^6–8^ Nuclear spins, in contrast, have a much smaller magnetic moment and require excitation by radiofrequency (RF) fields, which demand a substantially higher power (hundreds of W to kW). Their greater isolation from the environment makes them more robust and yields significantly longer coherence times, extending from tens of ms to seconds.^9–11^ Both types of spins can be coherently manipulated using magnetic resonance techniques to generate a superposition of states,^12^ which is an essential requirement for QIP.^13^

The separate manipulation of nuclear and electronic spins has found a variety of applications in QST. For instance, RF pulse sequences similar to those used in NMR spectroscopy have been exploited to implement the factorization of integer numbers,^14,15^ encode Grover’s algorithm into an electrically detected single-molecule transistor based on TbPc_2_ (*I* = 3/2),^16,17^ and build a molecular quantum processor.^18,19^ Likewise, coherent manipulation^7,10^ and the realization of adiabatic quantum computing schemes^20^ have been successfully demonstrated using MW radiation and EPR techniques. In this context, the transfer of spin coherence between electronic and nuclear spins would allow for leveraging advantages of both. Specifically, electronic spins can serve for fast initialization and/or readout, while the longer coherence lifetime of nuclear spins provides robust quantum memory.^21^ The combination of sequences of MW and RF pulses in Electron-Nuclear DOuble Resonance (ENDOR) techniques allows the simultaneous manipulation of electronic and nuclear spins, paving the way for the individual addressing and readout of the latter through the electronic spin echo. While these sequences achieve a transfer of population between electronic and nuclear spins, the coherence transfer among them requires a so-called Storage and Retrieval (SR) protocol consisting of more specific, combined MW and RF pulse sequences.

The coherence transfer via this protocol has been successfully implemented on single nitrogen vacancy (NV) centers in diamond to encode quantum registers^22^ and enhance their sensitivity as quantum sensors.^23^ Collections of NV centers in diamond (*S* = 1, *I* = 1), coupled to ^13^C nuclei (*I* = 1/2) in their environment, have been widely used for encoding solid-state quantum registers and memories.^24,25^ Similar milestone results have been achieved on ensembles of ^31^P electron donors in Si,^26^ rare-earth (Nd^3+^) ions in inorganic host crystals,^27^ and ^15^N atoms in fullerene.^28^ Furthermore, this protocol has been realized through the electrical readout of ^31^P in Si,^29^ and also in Mn^2+^-doped ZnO.^30^ These samples were typically measured at temperatures of 4–30 K on commercial EPR spectrometers equipped with a 3D microwave resonant cavity, using a dedicated (commercial or custom-made) resistive coil for the RF excitation. An alternative approach relies on the use of on-chip (superconducting) microwave resonators to address electronic spins at low temperature, eventually down to a few mK.^25,31^

Molecules carrying electronic or nuclear spins are emerging as an alternative to solid-state systems in QST,^32,33^ in particular for quantum computing^34^ and quantum sensing.^35–37^ Unlike spin defects in crystals, molecular architectures designed through a bottom-up approach offer tunable spin degrees of freedom^33^ and can be processed in a variety of ways, e.g., integrated into a nonmagnetic host matrix,^38,39^ attached to a surface for individual addressing by scanning probe methods,^40–43^ or precisely positioned close to a sensing target.^44–47^ They afford greater synthetic flexibility in the choice of nuclear species, enabling rational control of hyperfine multiplets by varying the ligand field, which can make electronuclear levels individually addressable. Shortened electronic coherence times relative to those of solid-state materials can be mitigated by optimizing the dilution rate or matrix geometry, and are outweighed by the benefits offered by molecular systems. Performing SR protocols exploiting these interactions in carefully engineered architectures would bring the success of experiments on solid-state systems to a molecular platform. These experiments largely benefit from the use of cryogenic temperatures, multifrequency MW/RF architectures (e.g., hybrid quantum circuits) to efficiently address molecular ensembles, and accurate alignment of the latter along static and oscillating (RF and MW) fields. Embedding molecular spins in a crystalline host matrix yields predictable, well-defined molecular orientations, reducing detrimental effects associated with the presence of the solvent. This approach greatly simplifies the experiment and makes molecular systems competitive for implementing coherent manipulation protocols.

In this work, we consider the heterobimetallic paddlewheel (PW) complex [VOPt­(SOCPh)_4_] (**1**) as a prototypical molecular qubit with multiple electronic and nuclear spin degrees of freedom and sufficiently long coherence times (11 μs in a frozen 1 mM solution in CD_2_Cl_2_/toluene-*d*_8_ at 10 K).^48^ Complex **1** contains a vanadyl ion (VO^2+^, *S* = 1/2) and a diamagnetic Pt^2+^ ion bridged by four nuclear spin-free monothiobenzoate ligands in a rigid Chinese lantern-like structure (Figure 1a). In this coordination environment, the unpaired electron is localized in the d_*xy*_ orbital of vanadium­(IV) and has the strongest hyperfine interaction with the *I* = 7/2 nucleus of ^51^V (natural abundance ∼100%). Together with the much weaker superhyperfine coupling with the *I* = 1/2 nucleus of ^195^Pt (natural abundance ∼34%), this creates a well-recognizable pattern in the EPR spectra of frozen solutions.^48^ Such an arrangement of electronuclear levels makes PWs an ideal molecular platform for encoding parallel algorithms with qudits^49,50^ or for performing nonconventional protocols for quantum sensing.^51^

**Figure 1:**
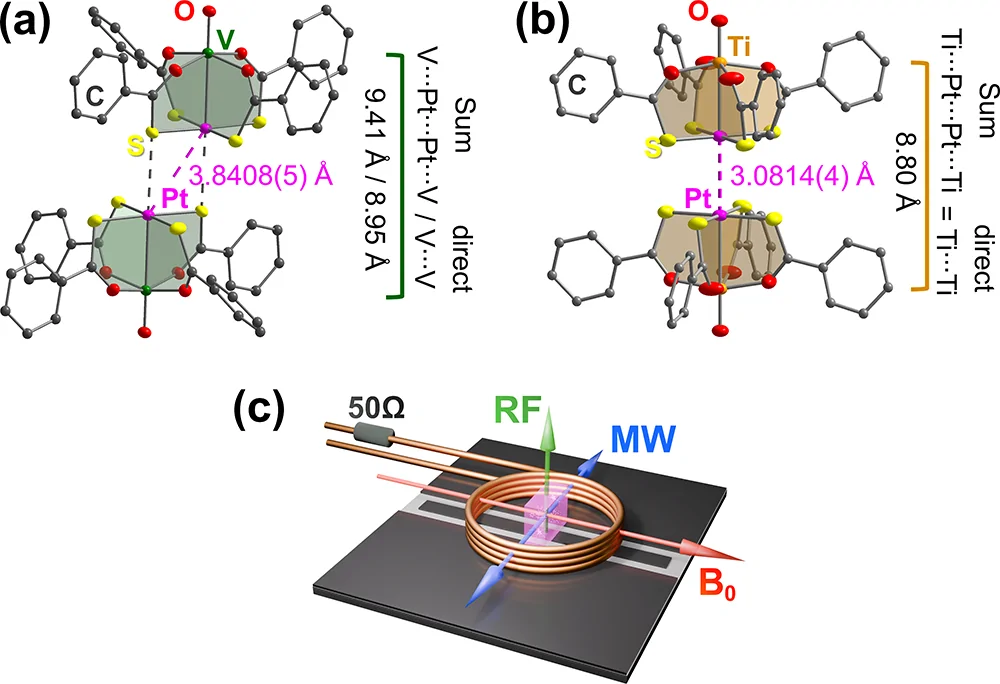
Molecular structure of the PW dimers in dichloromethane solvate **1**·DCM (a)^48^ and **2**·2THF (b); metals and heteroatoms are displayed as 50% probability ellipsoids, while carbon atoms are drawn in ball and stick style; hydrogens and disordered sulfur atoms with lower occupancy in (b) are omitted for clarity. (c) A superconducting YBCO coplanar resonator (black) used to deliver the MW pulses to the molecular spin crystal (purple). A copper coil is used to deliver RF pulses to the nuclei.

For our experiments on crystalline samples, we first developed a synthetic route to a diamagnetic analogue of **1**, [TiOPt­(SOCPh)_4_] (**2**), representing the first PW of this type containing a titanyl ion (Figure 1b). A single crystal of [Ti_1–*x*_V_*x*_OPt­(SOCPh)_4_]·2THF with *x* = 0.01 (**3**·2THF) was then embedded into a multifrequency setup based on a superconducting planar MW resonator combined with an RF copper coil,^52^ operating at a cryogenic temperature (Figure 1c). Compared with commercial magnetic resonance equipment, superconducting planar MW resonators^53,54^ are a scalable and convenient choice for on-chip MW experiments.^55,56^ Nuclear spins are addressed by a cylindrical millimeter-sized RF coil placed directly on the surface of the resonator.^36,52,57^ Our homemade heterodyne spectrometer, driven by an Arbitrary Waveform Generator,^36,58^ generates all necessary excitations, allowing us to perform both EPR and ENDOR spectroscopy on small crystals and to monitor both the in-phase and out-of-phase components of the detected echo signal. In this way, we characterized the spectroscopic features of **3**·2THF by continuous-wave (CW) EPR, Pulsed EPR, and Mims-ENDOR sequence.^59^ Finally, we performed a multifrequency SR sequence to show the possibility of transferring coherence between electronic and nuclear spins, thus experimentally demonstrating that the output spin echo quadrature signals (corresponding to *S*_*x*_ and *S*_*y*_) maintain the selected initialization after the whole protocol. Our results establish a foundation for joint electronic and nuclear spin manipulation in molecular spin centers.

## 2 Results and Discussion

### 2.1 Synthesis and Structure of the Pure and VO-Doped Titanyl Matrix

Significant differences in the chemical properties of vanadium and titanium account for the scarcity^7^ of diamagnetic titanyl analogues of vanadyl-based qubits. While vanadium­(IV) forms the robust and water-stable VO^2+^ cation, discrete titanyl complexes bearing a terminal Ti=O bond are rather exotic due to their high tendency to dimerize and oligomerize with the formation of Ti–O–Ti bridges or extended (−Ti–O)_*n*_ chains.^60^ With its higher oxophilicity, titanium­(IV) is also more prone to hydrolysis than vanadium­(IV), which complicates the synthesis of the PWs in an aqueous environment. For instance, the addition of polymeric TiO­(SO_4_)·2.3H_2_O to an aqueous solution of the [Pt­(SOCR)_4_]^2–^ metalloligand resulted in extensive hydrolysis and a negligibly low yield of **2** (<5%, identified by elemental analysis and ^1^H NMR).

Other readily available titanyl sources are monomeric K_2_[TiO­(C_2_O_4_)_2_]·2H_2_O, which may lead to mixed-ligand species, and dimeric [TiO­(acac)_2_]_2_,^61^ which was successfully employed in the synthesis of some titanyl phthalocyanine and porphyrin^62^ complexes (Hacac = acetylacetone). However, cleavage of its Ti–O–Ti bridges occurs under high-temperature conditions, which are incompatible with the aqueous medium needed for the metalloligand formation.

We successfully accessed pure **2** in 56% yield using [TiCl_2_(acac)_2_][Bibr ref63] as a source of titanium­(IV), which forms chemically available TiO^2+^ cations in situ (Figure 2a). Partial alcoholysis of [TiCl_2_(acac)_2_] to alkoxide complexes^64^ in MeOH was followed by simultaneous hydrolysis and assembly of the heterobimetallic structure by reaction with understoichiometric aqueous [Pt­(SOCPh)_4_]^2–^ (2.5:1). Compound **2** forms along with a number of byproducts displaying a similar solubility in organic solvents, including acac^–^ and other thiobenzoate derivatives (Figure S5). The layering technique used for the vanadyl^48,65^ analogue was ineffective for purification, since the titanyl PW precipitates along with some of the byproducts on contact with a “bad solvent” (*n*-hexane or Et_2_O). Instead, large crystals of pure **2**·2THF (up to 1 mm in size) were grown from an oversaturated THF solution by repeated heating and cooling cycles.^66^ Alternatively, the complex can be purified by short-column chromatography on silica gel using anhydrous chloroform as eluent. Two additional solvates were also isolated by recrystallization from toluene (Supplementary note 1).

**Figure 2:**
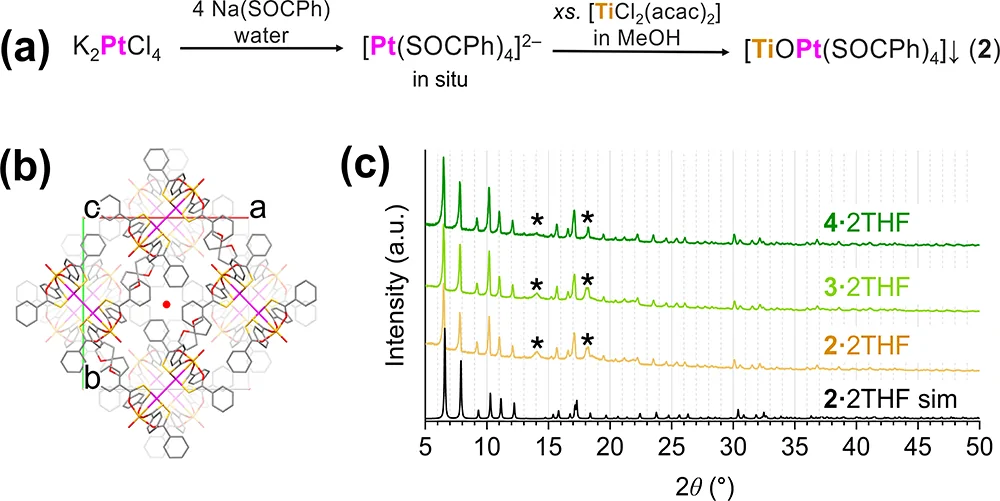
(a) Scheme of the synthesis of **2**. (b) Packing of **2**·2THF viewed along the *c* axis, drawn in capped sticks style; the 4-fold axis is indicated by a red dot. (c) Experimental PXRD patterns of freshly crystallized samples collected at 298 K (peak intensities were normalized in counts per second). The black line at the bottom represents the simulated PXRD pattern of **2**·2THF, calculated from its single-crystal XRD (SXRD) structure collected at 200 K (CCDC 2477890). The peaks marked with asterisks are due to addenda (see Experimental section).

Compound **2**·2THF crystallizes as square-shaped yellow blocks belonging to the tetragonal *I*4̅*c*2 space group, with half a titanyl complex in the asymmetric unit. The PWs assemble into staggered dimers supported by Pt···Pt contacts (Figure 1b), with the Pt, Ti, and O­(oxo) atoms positioned on a 2-fold axis perpendicular to the tetragonal axis *c* (Figure 2b). Other solvates (Supplementary note 1) do not display such a fortunate arrangement of molecules. The metallophilic Pt···Pt contact is slightly shorter than found in the vanadyl analogues (3.08 vs 3.17–3.23 Å),^67^ while the larger ionic radius of titanium­(IV) vs vanadium­(IV) leads to an expanded metal-oxo separation (1.63 vs 1.58 Å). We note that **1** forms a monotetrahydrofuran solvate **1**·THF (identified by unit cell check), which is isomorphous to **1**·DCM^48,65^ and contains PW dimers with a “square” structure supported by two Pt···S interactions (Figure 1a).^68^ However, since these dimers dissociate in organic solutions, as shown by ^1^H diffusion ordered spectroscopy (DOSY) (Supplementary note 2, ref 48), and dimerization is controlled by a shallow energy surface,^67^ the different structural preferences of Ti and V derivatives are not critical. Indeed, crystalline phases [Ti_1–*x*_V_*x*_OPt­(SOCPh)_4_]·2THF with nominal concentrations *x* = 0.01 (**3**·2THF) and 0.10 (**4**·2THF) isomorphous to the pure titanyl matrix **2**·2THF (Figure 2c) were obtained by cocrystallization of mixed vanadyl and titanyl PWs from THF. The effective concentration of vanadyl ions was determined by inductively coupled plasma mass spectrometry (ICP–MS) as *x* = 0.0068(1) and 0.090(2) for **3**·2THF and **4**·2THF, respectively.

**4**·2THF (*x* = 0.10) already contains magnetically independent *S* = 1/2 spins, as shown by the simple Brillouin-like behavior found in DC magnetic measurements (Figure S9). Moreover, while crystalline **1**·DCM has no measurable slow magnetic relaxation,^48^ presumably because of the sizable intra- and interdimer magnetic coupling, **4**·2THF exhibits a detectable out-of-phase component of the AC susceptibility, albeit only under a nonzero DC field (*H*). Its spin–lattice relaxation time τ­(*T*,*H*) reaches up to 120 ms at *T* = 1.85 K and *H* = 10 kOe (see Supporting Information, Figures S10–S13). Much lower doping levels could not be investigated by DC/AC techniques due to the small spin value and insufficient sensitivity.

### 2.2 Magnetic Resonance Results

#### 2.2.1 Single-Frequency Magnetic Resonance

Single-frequency CW-EPR was preliminarily used to characterize **3**·2THF, which provides an optimal balance between the electronic phase memory time (*T*_m_) and the intensity of the spin echo signal. A single crystal was oriented with its tetragonal *c* axis aligned with the static magnetic field **B**_0_ (Figure 1c) to probe the direction orthogonal to the V–Pt vector for all molecules (Figures 2b, S14, Supplementary video).

Scanning the static magnetic field at a fixed probe frequency (∼7.4 GHz) yielded an eight-line pattern (Figure 3a), characteristic of the hyperfine interaction between the electronic (*S* = 1/2) and nuclear (*I* = 7/2) ^51^V spins of vanadium­(IV).^48,66,69^ The shape of the spectrum confirms no detectable dimerization or aggregation of vanadyl PWs within the matrix. Each EPR line has two smaller satellite peaks arising from the superhyperfine coupling with the *I* = 1/2 nuclear spin of ^195^Pt. This was corroborated by the EasySpin^70^ fit performed starting from the spin Hamiltonian parameters found on frozen solutions (Supplementary note 3).^48^ The fit, with all other parameters fixed, gave *g*_*x*,*y*_ = 1.995 and *A*_*x*,*y*_ = −204.3 MHz; the negative sign of the hyperfine tensor **A** was assigned based on literature works involving VO^2+^.^71–73^ A Zeeman diagram demonstrating the corresponding transitions is shown in Figure 3b.

**Figure 3:**
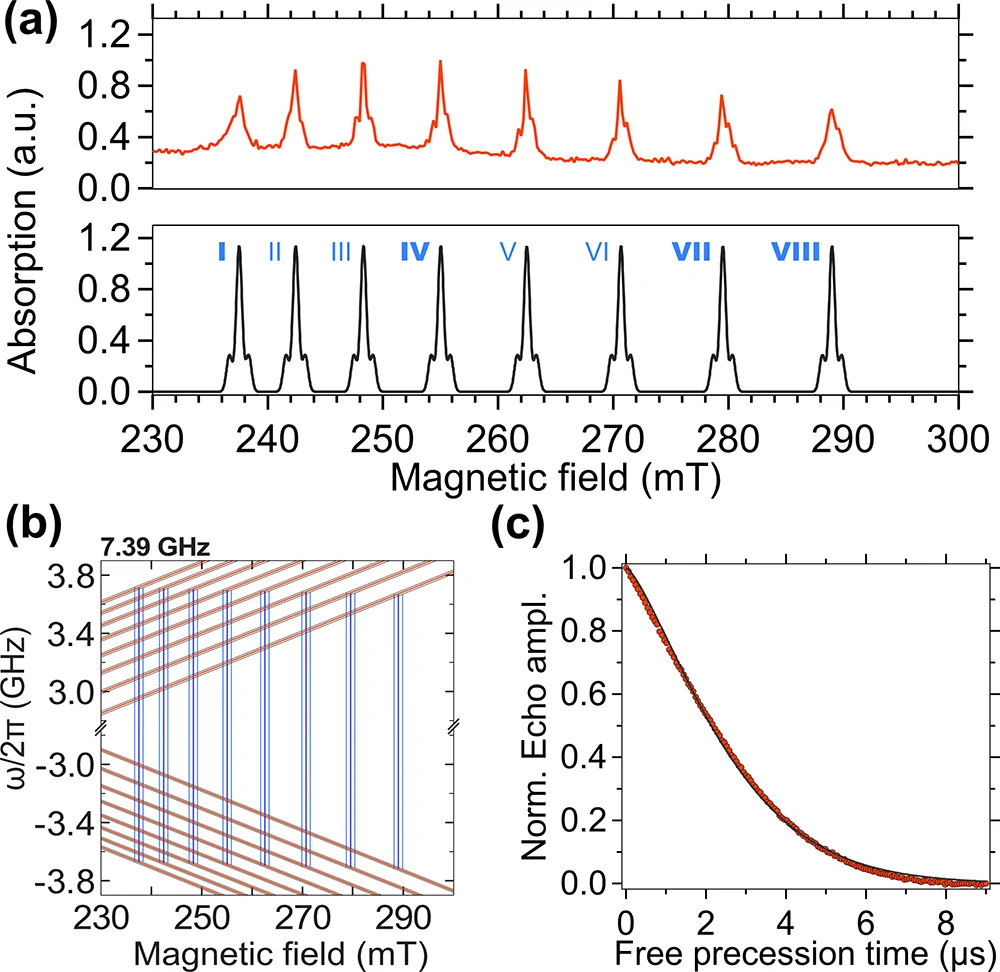
(a) CW-EPR spectrum of **3**·2THF measured with a YBCO coplanar resonator at 3 K (red). The position of the eight absorption peaks was confirmed by an EasySpin fit (black line). Electronic transitions are labeled with Roman numerals from low to high field. The transitions used in this work are indicated with bold text. (b) Zeeman diagram of the hyperfine electronuclear spin levels of VO^2+^, obtained from an EasySpin fit of the CW-EPR spectrum. The spin levels for molecules containing nonmagnetic Pt isotopes (red) and ^195^Pt (black) are shown alongside their allowed electronic transitions (blue and light blue, respectively). (c) Decay of the echo amplitude. The experimental data (red) are fitted with eq 1 (black).

The electronic phase memory time of **3**·2THF was studied using Hahn echo sequence at 3 K.^74^ The echo amplitude decay shown in Figure 3c was measured for the electronic transition with the highest absorption (*B*_0_ = 255 mT) and was fitted with a stretched exponential function, eq 1:

1 $$A\left(t\right)=A\left(t_{0}\right)\cdot e^{-{\left(\frac{t-t_{0}}{T_{m}}\right)}^{\beta }}$$

where *t* is the total free precession time (i.e., 2τ), *t*_0_ is the starting time, *A*(*t*_0_) is the amplitude at *t*_0_, and β is the stretching parameter. The fit converged to β = 1.4 and *T*_m_ = 2.8 μs, determining the time scale for coherent manipulations on electronic spins. The stretching parameter value may reflect the spin diffusion effect caused by the ligand protons and Pt nuclei near the vanadyl center.^75,76^

#### 2.2.2 Electron-Nuclear DOuble Resonance (ENDOR)

All ENDOR measurements were performed by means of Pulsed Mims-ENDOR,^59^ consisting of three MW π/2 pulses combined with a single RF π pulse (Figure 4a). The latter excites a resonant nuclear transition during the electronic free precession time, causing transfer of polarization to the nuclear spin, which also affects the amplitude of the electronic spin echo. This variation in amplitude, recorded as a function of the RF pulse frequency, is expressed in terms of ENDOR efficiency (*F*_ENDOR_), defined by eq 2:

2 $$F_{ENDOR}=\left|\frac{A_{echo}^{ON}-A_{echo}^{OFF}}{2A_{echo}^{OFF}}\right|$$

where *A*_echo_^ON^ and *A*_echo_^OFF^ are the amplitudes of the spin echo when the RF pulses are ON or OFF, respectively.^12^ Peaks in *F*_ENDOR_ appear when a nuclear resonance is met, allowing for the location of the nuclear transition positions. For this purpose, the Davies-ENDOR^77^ sequence also proved successful and gave similar results (Figure S18), but at a slightly lower resolution than Mims-ENDOR.

**Figure 4:**
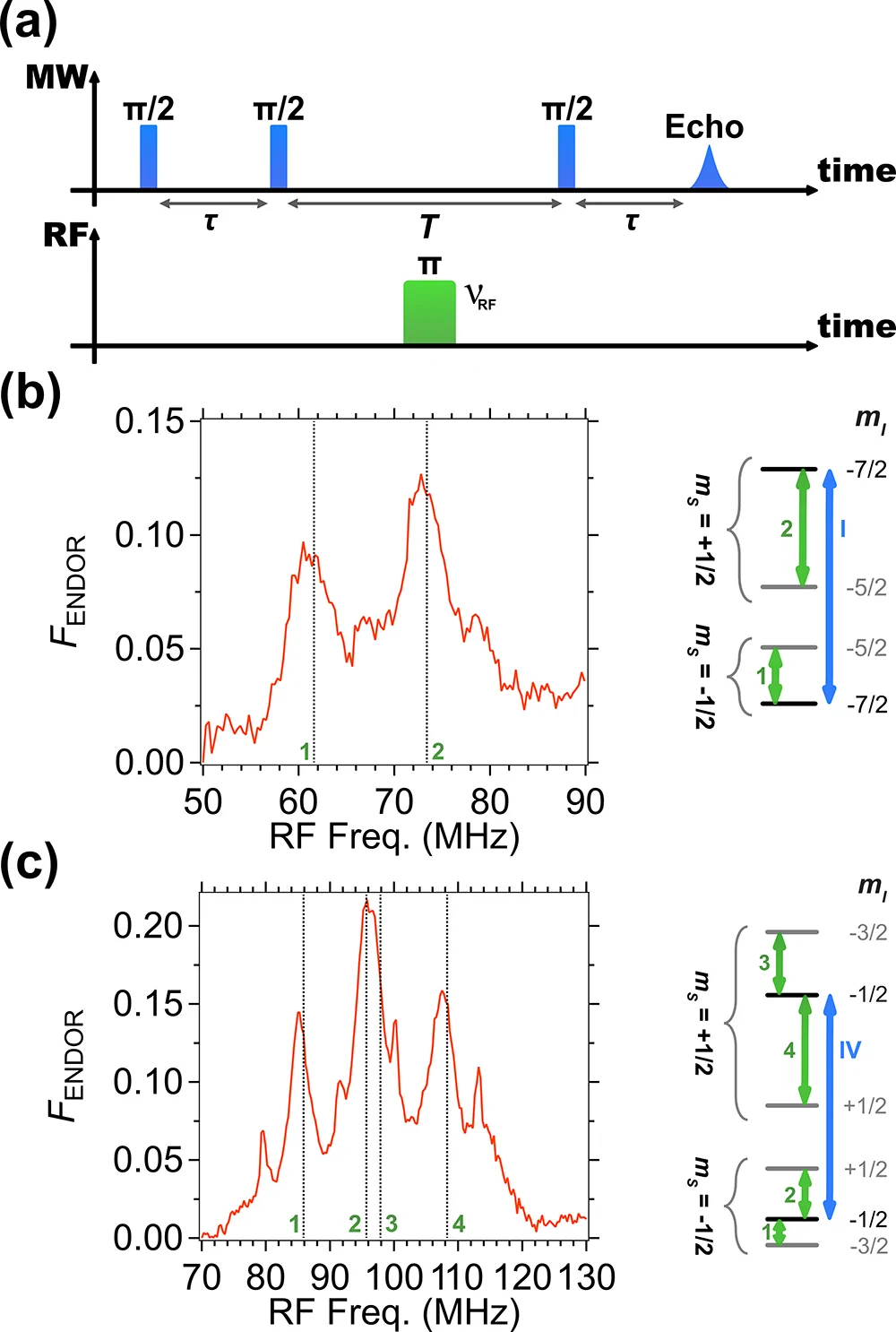
(a) Mims-ENDOR sequence. The spin echo is monitored at each RF value (ν_RF_) and plotted as *F*_ENDOR_. (b) Mims-ENDOR spectrum taken at 3 K and *B*_0_ = 237.7 mT (line I in Figure 3a). (c) Mims-ENDOR spectrum taken at 3 K and *B*_0_ = 255.0 mT (line IV in Figure 3a). A sketch for the electronic (blue) and nuclear (green) transitions is shown for (b,c), with calculated nuclear transition frequencies (black vertical dashed lines) numbered with Arabic numerals accordingly in each spectrum.

For *B*_0_ fixed at 237.7 mT (line I in Figure 3a), in resonance with the electronic transition corresponding to *m*_*I*_ = −7/2, two main peaks appear in the Mims-ENDOR spectrum (Figure 4b), as expected from an EPR transition located at the lowest or the highest resonant magnetic field value,^12^ which is also consistent with previously reported ENDOR spectra on VO^2+^.^73,78^ At *B*_0_ = 255.0 mT (line IV in Figure 3a), which is resonant with the electronic transition corresponding to *m*_*I*_ = −1/2, three peaks appear (Figure 4c), with the central one resulting from two overlaid nuclear transitions close in energy. The positions of the ENDOR peaks for all nuclear transitions match well with the energy differences obtained from the Zeeman diagram of Figure 3b (see also Figure S16, Table S3). According to additional simulations (Supplementary note 4), the *F*_ENDOR_ peaks (Figure S17, Table S4) arising from the superhyperfine splitting with a fraction of magnetic ^195^Pt are hidden within the line width of the main ^51^V peaks in Figure 4b,c. This suggests that, under our experimental conditions, individual transitions on ^195^Pt cannot be properly resolved using the applied MW/RF protocols. Additional Mims-ENDOR measurements, performed around the ^1^H resonance frequency (Figure S15), show the expected behavior also for this nucleus. Overall, these results show the possibility of addressing individual ^51^V nuclear transitions and reading them out using electronic spin echo.

#### 2.2.3 Storage and Retrieval (SR) of Coherence between Electronic and Nuclear Spins

SR protocol consists of a specific set of resonant MW and RF pulses. Figure 5a outlines the pulse sequence, and Figure 5b illustrates the evolution (red arrow and circles) of the spin coherence (represented by the blue or green double arrows) under the action of the pulses.^26,27^ Figure 5c gives details on the storage block.

**Figure 5:**
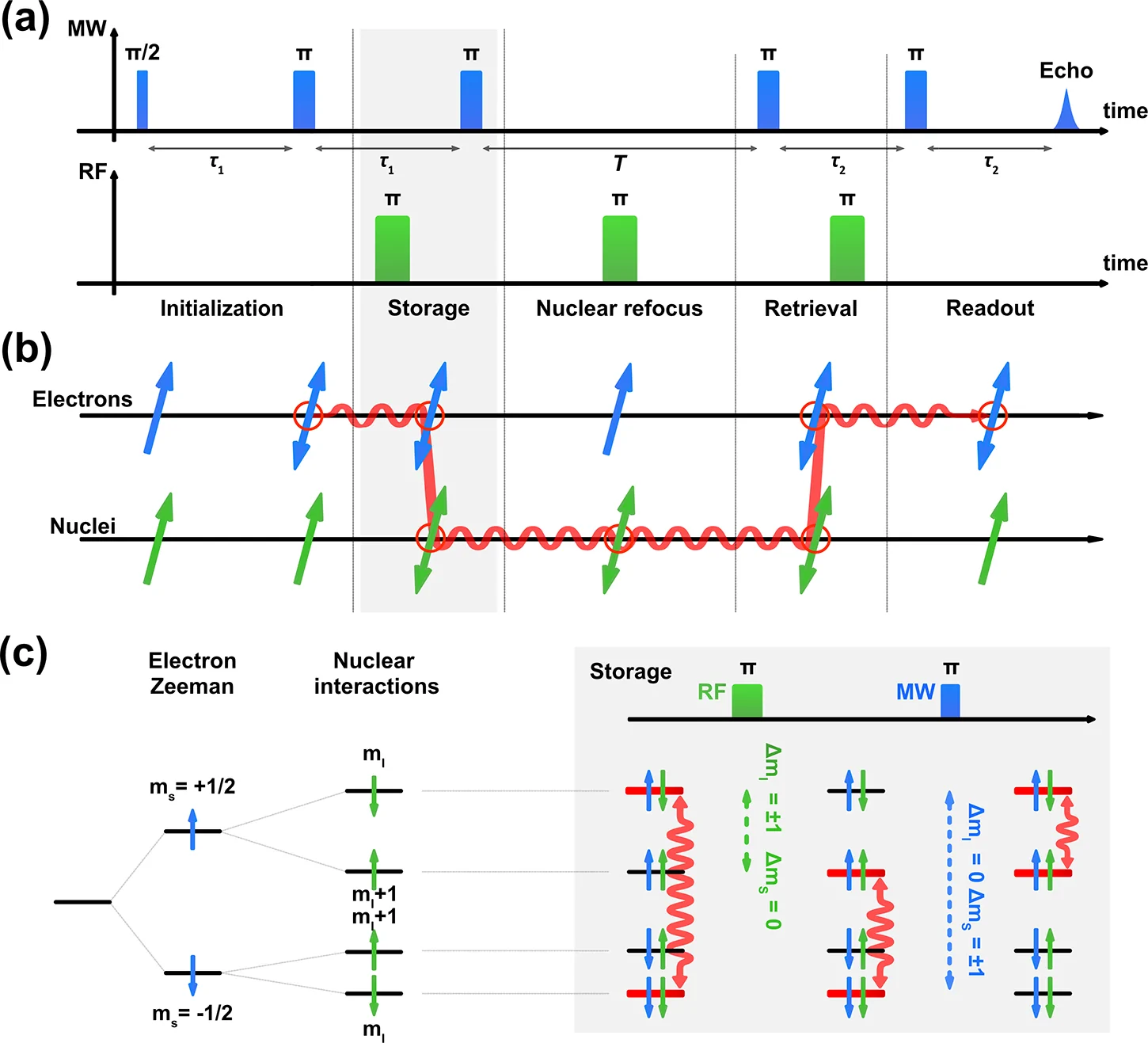
(a) Sketch of the full multifrequency SR pulse sequence. (b) Sketch of the evolution of coherence (red wavy arrow) during the sequence. The coherence (double arrow and red circle) generated between the electrons (blue arrows) is transferred to the nuclei (green arrows) and then retrieved at the end of the sequence. (c) Details of the storage block in (b). Two electronic *m*_*s*_ = ± 1/2 levels of **1**, and only two of the corresponding hyperfine-split levels (*m*_*I*_, *m*_*I*±1_) are represented for better clarity. Arrows represent the electronic (blue) and the nuclear (green) spins at each level. The red wavy arrows represent the coherence between two levels and its evolution under the action of the MW (blue) and RF (green) π pulses.

The SR protocol operates as follows. During the initialization phase, the first MW π/2 pulse and the following refocusing MW π pulse generate coherence on the electronic transition (first blue double arrow). The phase of the first π/2 pulse determines the phase of the output electronic spin echo.^79^ Next, in the storage block, two subsequent pulses are applied to transfer and store the electronic coherence into the nuclear spins (first linked green and blue double arrows). Specifically, as shown in Figure 5c, an RF π pulse first inverts the polarization of the resonant nuclear transitions involving levels *m*_*I*_ and *m*_*I*+1_ of the *m*_*S*_ = +1/2 manifold. Then, an MW π pulse inverts the polarization of the *m*_*S*_ = ± 1/2 transition at a fixed *m*_*I*_. After the MW pulse, the coherence involves only the nuclear *m*_*I*_ and *m*_*I*+1_ states in Figure 5c (second green double arrow in Figure 5b).

At this point, in a different pulse sequence, measuring an electronic spin echo would yield results similar to those from a standard ENDOR sequence (i.e., an electronic spin echo altered by the nuclear polarization transfer, as in Figure S19). During the nuclear refocus step, an additional RF π pulse can also be applied at the center of the sequence to maintain the nuclear coherence. When implementing QIP protocols within this mixed electronic-nuclear spin approach, this simple pulse can be replaced with more complex (eventually multifrequency) RF pulse sequences.^9,49,50^

In our experiment, a transfer of coherence (along with its initial phase) from nuclear back to electronic spins is finally achieved using a reversed couple of pulses, i.e., an MW π pulse followed by an RF π pulse (retrieval phase in Figure 5a, second couple of green and blue double arrows in Figure 5b). The final refocusing MW π pulse generates an electronic spin echo used as a readout (last blue double arrow).

Before applying the full SR protocol, we monitored the amplitude and phase of the electronic spin echo by stepwise changing the phase of the MW π/2 pulse, θ_π/2_, of a Hahn echo sequence (with a fixed interpulse delay τ = 1.8 μs, Figure 6a).^79^ This provided a reference for the echo quadrature signals corresponding to the *S*_*x*_ and *S*_*y*_ electronic spin components, which coherently followed the MW π/2 initialization (Figure 6b). The duration of this purely electronic process is limited by *T*_m_. In fact, for interpulse delays much longer than *T*_m_, the echo amplitude drops to zero, and the echo components cannot be retrieved (see Figure S20).

**Figure 6:**
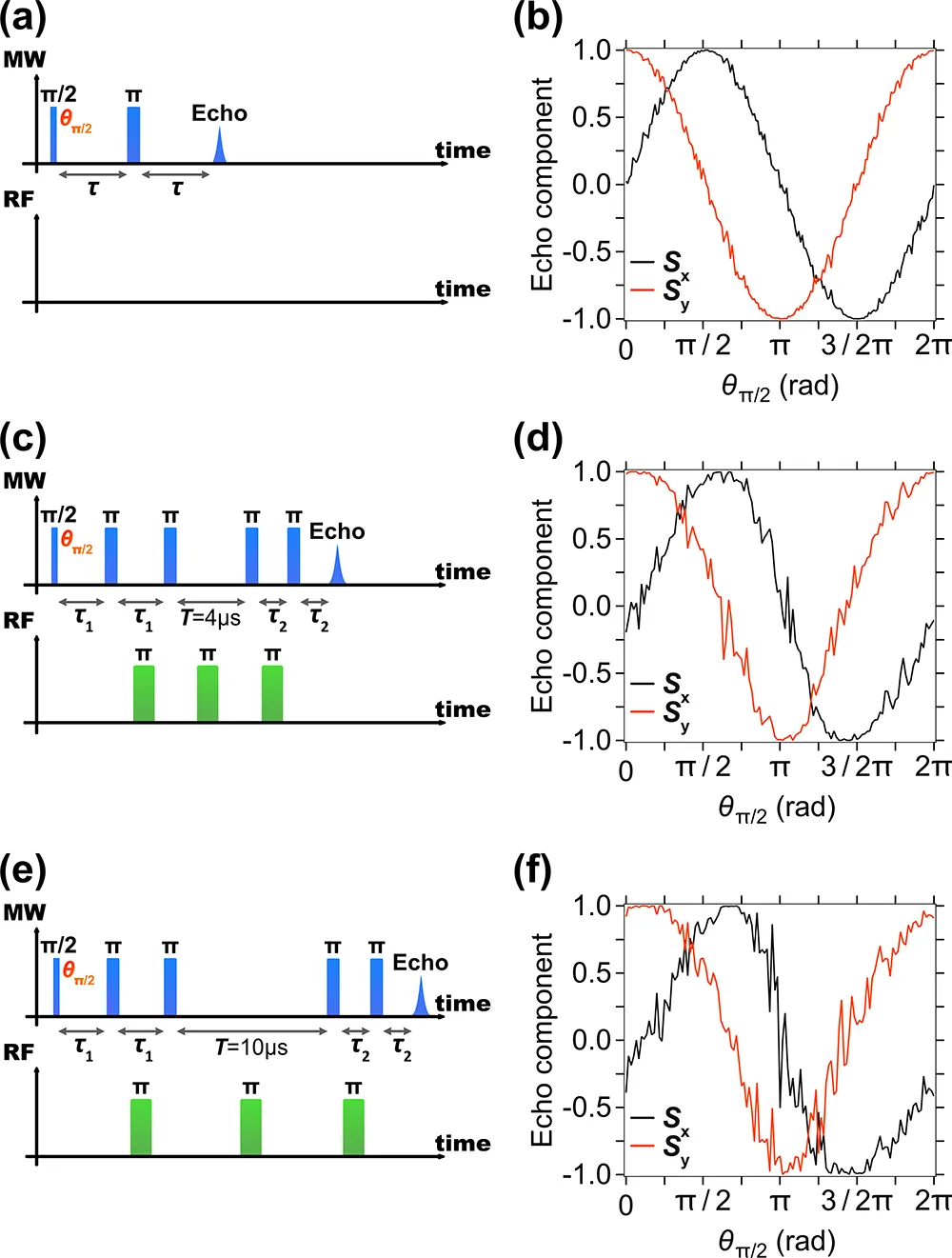
(a) Hahn echo sequence used to obtain the reference signals for the SR protocol. (b) Echo quadrature signals, corresponding to the *S*_*x*_ and *S*_*y*_ components of the electronic spin, measured with the protocol in (a) as a function of the phase of the first MW π/2 pulse. (c,e) Sketch of the full SR protocol used (see Figure 5). The delay between the first and the last group of MW pulses, *T*, is set to 4 or 10 μs. (d,f) Echo quadrature signals extracted from the electronic spin echo obtained with the protocol in (c,e) as a function of θ_π/2_ for the electronic transition IV (see Figure 3a) and the nuclear transition 1.

Then, we tested the full SR sequence on the electronic transition IV and nuclear transition 1 (Figure 4c) with delays *T* = 4 μs, τ_1_ = τ_2_ = 1.6 μs (see Figure 6c). Nonsymmetric τ_1_ and τ_2_ can be chosen to avoid the overlap of unwanted echoes with the measured one. The quadrature signals of the electronic spin echo (see Figure 6d) match well the behavior of the reference signal, demonstrating that the spin coherence survives during the entire protocol (the total free precession time is *t*_tot_ = 2τ_1_ + 2τ_2_ + *T* ∼ 10 μs > *T*_m_). Repeating the same protocol with a longer central delay, *T* = 10, 30, and 50 μs, gave similar results (Figures 6e,f, S21). This implies that the correct behavior of the *S*_*x*_ and *S*_*y*_ electronic spin components is retrieved after a time *t*_tot_ much longer than the electronic *T*_m_. Additional results, obtained by repeating the SR protocol as in Figure 6f but with all RF pulses turned OFF, showed that the spin echo components no longer match the behavior of the reference signal (Figure S20), confirming the key role of the nuclear spin degrees of freedom in the sequence.

To estimate the efficacy of the SR protocol, we introduced a fidelity-like parameter, the *in-plane fidelity*, which quantifies the deviation of the output echo phase from the input value (Supplementary note 5). The sustained values of this parameter across extended storage times confirm that quantum information can be reliably preserved even over long storage intervals. Altogether, our findings experimentally support the potential of joint use of electronic and nuclear states of molecular systems for QIP.

## 3 Conclusions

In this work, we designed a crystalline matrix of the first titanyl paddlewheel, [TiOPt­(SOCPh)_4_]·2THF, and doped it with variable molar fractions (0.01, 0.10) of [VOPt­(SOCPh)_4_]. Close molecular similarity leads to homogeneous cocrystallization of PWs with the host structure retained across the examined concentration range. DC/AC magnetic studies showed independent behavior of *S* = 1/2 spins at the highest explored concentration, with the spin-lattice relaxation time reaching up to 120 ms at *T* = 1.85 K and *H* = 10 kOe.

Using a superconducting YBCO coplanar MW resonator, combined with an RF copper coil and a homemade heterodyne setup, we performed multifrequency CW-EPR and pulsed ENDOR spectroscopy on a single crystal of [Ti_0.99_V_0.01_OPt­(SOCPh)_4_]·2THF oriented to probe all molecules in the perpendicular direction. Well-resolved CW-EPR spectra enabled addressing of individual hyperfine lines with MW pulse sequences. Nuclear spin transitions of ^51^V were excited individually through Mims-ENDOR spectroscopy. Finally, the transfer of phase coherence between electronic and nuclear spins was tested by performing the SR protocol. Specifically, the detection of electronic spin echo components, following multifrequency MW/RF manipulation, demonstrated that coherence remains recoverable after a 30 μs free precession period. These durations are significantly longer than the 2.8 μs intrinsic electronic Hahn-echo memory time, so the coherence time increases by more than an order of magnitude compared to purely electronic storage. Replacing the central refocusing RF pulse with a pulse train will enable the implementation of more complex quantum processing algorithms, facilitated by simplified encoding and readout through electronic spin echo.

The ability to simultaneously control both electronic and nuclear spin states has already proven crucial for qubit-qudit coherent manipulation^1,22^ and quantum sensing.^23,80^ While these capabilities have been widely demonstrated in solid-state platforms,^26–28^ the next frontier lies in exploiting intrinsic features of molecular spin centers, which previously relied on electronic or nuclear spins alone.^58,81^ In this context, our work bridges the gap and opens up novel molecular routes for quantum technologies.

## 4 Experimental Section

### 4.1 Details on Synthesis

#### 4.1.1 General Methods

All solvents were dried over activated 4 Å molecular sieves except for MeOH (3 Å). DCM, toluene, *n*-hexane, and MeOH used for normal operations were not further purified. Chloroform (containing EtOH as stabilizer) was washed three times with water, dried over Na_2_SO_4_, passed through a short column of basic alumina, and stored with the addition of amylene. THF was dried as previously described: first distilled over KOH, then over Na/benzophenone under a nitrogen atmosphere,^82^ and stored in a nitrogen-filled Schlenk vessel. All solvents used for glovebox operations under nitrogen, including CD_2_Cl_2_ (99.8 atom % D), were degassed by three freeze–pump–thaw cycles. CDCl_3_ (99.8 atom % D) was passed through a layer of basic alumina and stored at 4 °C. The water content in commercial VOSO_4_·*n*H_2_O was determined by elemental analysis (EA) as *n* = 3.6. K_2_PtCl_4_ was used as received. NaSOCPh was prepared by reacting HSOCPh (92%, contaminated with benzoyl disulfide according to ^1^H NMR) with NaHCO_3_ in water, and its purity was checked by ^1^H NMR in D_2_O, EA, and IR spectroscopy. [VOPt­(SOCPh)_4_] (**1**) was obtained by a known procedure.^48,65^ Air-sensitive [TiCl_2_(acac)_2_] was prepared following a literature procedure^63^ without recrystallization, utilizing standard Schlenk techniques, and its purity was confirmed by EA and ^1^H NMR.

EA (CHN) was performed on a ThermoFisher Scientific Flash 2000 analyzer. IR spectra were measured on a Jasco 4700 FT-IR spectrometer (ATR mode, resolution 1 cm^–1^). ^1^H NMR spectra were acquired at 298 K on ∼10 mM solutions in CD_2_Cl_2_ or CDCl_3_ using a Bruker Biospin Avance 400 spectrometer. Chemical shifts (δ, ppm) are referenced to the residual proton resonances of the solvent (^1^H CD_2_Cl_2_: δ = 5.32; CDCl_3_: δ = 7.26; ^13^C CD_2_Cl_2_: δ = 54.0; CDCl_3_: δ = 77.2)^83^ or to TMS as an internal standard. ^1^H DOSY NMR measurements (Supplementary note 2) were carried out with a ledbpgp2s sequence (Bruker library) using bipolar gradient pulses.^84^ The diffusion time (big delta) and the gradient length (small delta) were tuned to 0.07 s and 900 μs, respectively, using the monodimensional sequence ledbpgp2s1d (Bruker library). The signal decay was fitted with a single exponential function using Bruker Dynamic Center software (version 2.8.3). Diffusion coefficients were normalized using TMS as an internal reference following the procedure described by Stalke et al.^85^ Alternatively, the residual proton signal of CD_2_Cl_2_ was used as a reference for the external calibration curves (ECCs) developed by Byers et al.^86^ The absolute errors on estimated molecular weights were calculated as reported by Stalke et al., who showed that the uncertainties on ECC’s parameters largely determine these errors.^87^ The electronic absorption spectrum (Figure S7) was collected on a 7.9 × 10^–4^ M solution in DCM using a Jasco V-770 UV–vis–NIR spectrometer operating in double-beam mode (optical path length *l* = 0.1 cm). The ICP–MS measurements were conducted with an iCAPTQ spectrometer (Thermo Fisher Scientific) equipped with an autosampling system for sample introduction (ESI FAST 2DX) and a microflux (400 μL min^–1^) ESI nebulizer (PFA). The analyses were conducted in triple-quadrupole mode using O_2_ as the reaction gas. The samples were digested using HNO_3_ (65% w/w, Suprapur) and H_2_O_2_ (30% w/w) and diluted using a solution of 2% v/v HNO_3_ (prepared using bidistilled water obtained with a Milli-Q IQ 7000 system). Five analytical measurements were performed for each standard, blank, and sample. All the samples were measured in duplicate. Sc and Tb solutions were used as internal standards.

#### 4.1.2 Synthesis of [TiOPt­(SOCPh)_4_] (**2**)

A solution of K_2_PtCl_4_ (200.4 mg, 0.4828 mmol) in 5 mL of water was added dropwise to a solution of NaSOCPh (310.5 mg, 1.935 mmol, 4 equiv) in 20 mL of water. In the course of 2 h, the color of the reaction mixture changed from pink to yellow, and some brownish precipitate formed, which was removed by filtration. [TiCl_2_(acac)_2_] (229.0 mg, 0.7224 mmol, 1.5 equiv) was split into three equal portions, and each of them was treated with a minimal amount of MeOH (1.5–2 mL) before it was added dropwise to the Pt solution, with an interval of 5 min between successive portions. The addition of the first portion caused the immediate precipitation of a yellow powder, which visually thickened, forming small agglomerates, after the addition of the second portion. After 30 min, further [TiCl_2_(acac)_2_] (150.2 mg, 0.4738 mmol, 1 equiv), split into two equal portions, was added to the reaction mixture in the same manner. The reaction was allowed to proceed for 2.5 h, after which the precipitate was collected on a G4 filter, washed extensively with water and 3 mL of MeOH, and dried in a vacuum oven at 70 °C overnight, yielding 414.6 mg (389.6 mg theoretical yield) of the raw product. The filtrates, colorless or pale-yellow during filtration, produce fine precipitates of TiO_2_/TiO­(acac)_2_/other mixed species upon standing, and no further product can be collected after the isolation of the main batch.

*Note.* The stepwise dosing of prealcoholysed [TiCl_2_(acac)_2_] (2.5 equiv, split in five portions) is crucial to maximize the product yield; the addition of 1–1.5 equiv split into portions of 0.5 equiv each reduces the yield by 12–20%.

**Purification procedure A.** The precipitate from the filter was collected with 20 mL of THF, and the solvent was evaporated. The solid, consisting of yellow **2** and more-soluble brown byproducts, was washed with ice-cold THF (3 × 1.5 mL) and dried in vacuo, giving 282.9 mg of crude product. Stepwise crystallization from 2 mL of THF was carried out in the glovebox by cycling heating to 60 °C and cooling to room temperature over 2 weeks, with THF changed five times. The resulting yellow crystalline product was dried in vacuo (240.5 mg, 0.2679 mmol, 56%). EA for **2**·1.25THF (897.80): C 44.15, H 3.37%; found C 43.94, H 3.68%. The content of THF decreases with grinding or long-term storage.

**Purification procedure B.** 309.2 mg of the raw product (obtained using 1.5 equiv of [TiCl_2_(acac)_2_]) was subjected to fast chromatography on a short silica column (SiO_2_ chromagel 60 Å, 35–70 μm, column ⌀ = 1.5 cm, column length = 10 cm), using pure CHCl_3_ as eluent. Evaporation of the solvent with subsequent recrystallization from THF gave 180.4 mg (0.2051 mmol, 42%) of the pure product. EA for **2**·THF (879.77): C 43.69, H 3.21%; found C 43.27, H 3.54%.

^1^H NMR (400 MHz, CDCl_3_): δ 8.34 (dd, *J* = 8.5, 1.2 Hz, 8H, *o*-C_6_ *H*_5_), 7.64–7.51 (m, 4H, *p*-C_6_ *H*_5_), 7.49–7.33 (m, 8H, *m*-C_6_ *H*_5_). IR (Figure S6, ν̃_max_, cm^–1^): 1556 m, 1502 s, 1456 m, 1430 m, 1367 w br, 1326 w, 1299 w, 1211 s, 1172 s, 1064 m, 1040 w, 999 w, 958 s, 946 s, 911 m, 862 m, 850 m, 843 m, 820 m, 810 w, 795 w, 771 m, 722 m, 694 m sh, 683 s, 646 m, 615 w, 605 w, 599 w, 580 m, 572 m, 555 w br, 533 m, 504 s, 494 m sh, 447 w 438 w.

When **2** was recrystallized from toluene in the same way as **1** in ref 67, two solvatomorphic modifications were identified: needle-like **2**·0.5tol, formed in minor amounts above the solution level, and thick plates of **2**·0.75tol (main) under the mother liquor (Supplementary note 1). The mother liquor was decanted, the crystalline product was washed with *n*-hexane (6 × 1 mL) and dried in a vacuum. After thorough drying, EA finds a reduced toluene content. For **2**·0.5tol (853.73): C 44.32, H 2.83%; found C 44.47, H 2.95%. ^1^H NMR (Figure S2, 400 MHz, CD_2_Cl_2_): δ 8.30 (dd, *J* = 8.5, 1.2 Hz, 8H, *o*-C_6_ *H*_5_), 7.69–7.52 (m, 4H, *p*-C_6_ *H*_5_), 7.53–7.35 (m, 8H, *m*-C_6_ *H*_5_). ^13^C NMR (Figure S3, 101 MHz, CD_2_Cl_2_): δ 215.77, 138.65, 134.92, 129.63, 129.11, 128.72.

#### 4.1.3 Solid Dilution Experiments

The crystallizations were performed in a nitrogen-filled glovebox to avoid trace moisture. Solutions were handled avoiding the use of metallic tools.

[Ti_0.99_V_0.01_OPt­(SOCPh)_4_]·2THF (**3**·2THF): to **2** (0.04949 mmol) dissolved in 4 mL of DCM, a 1.53 mM DCM solution of **1** (0.325 mL, 0.497 × 10^–3^ mmol, nominal 1.00 mol % V) was added. After evaporation to dryness, 0.7 mL of THF was added, and the crystals were grown by repeated heating and cooling cycles. 0.68(1) mol % of V (ICP–MS).

[Ti_0.9_V_0.1_OPt­(SOCPh)_4_]·2THF (**4**·2THF): **2** (0.0640 mmol) and **1** (0.00702 mmol, nominal 9.88 mol % V) were dissolved in 4 mL of DCM. After evaporation to dryness, 1 mL of THF was added, and the prominently green crystals were grown by repeated heating and cooling cycles. 9.0(2) mol % of V (ICP–MS).

#### 4.1.4 X-ray Crystallography

A Bruker-Nonius X8APEX diffractometer, equipped with a Mo–Kα generator, an area detector, and a Kryoflex cryostat, was used to collect SXRD data at 200 K on **2**·2THF, and at room temperature on **2**·0.5tol and **2**·0.75tol (Table S2). Crystals were immersed in one of the components of an epoxy resin or in NVH oil (Jena Bioscience) and mounted on a MiTeGen Microloop or on the flame-sealed tip of a glass capillary. APEX2 v1.0-22 software was used to collect frames, while data reduction was performed with SAINT v7.06A program and was followed by scaling and multiscan absorption correction using SADABS v2.10.^88^ The structures were solved and refined following standard procedures available in WINGX v2020.2 suite^89^ (SIR92 software^90^ for direct methods and SHELXL-2018/3 program^91^ for full matrix least-squares refinement on *F*_o_^2^). Unless otherwise noted, all non-hydrogen atoms were treated anisotropically. Isotropic displacement parameters for H atoms were constrained to *U* = 1.5*U*_eq_(C) for methyl groups and *U* = 1.2*U*_eq_(C) for the other H atoms.

In **2**·2THF, the disordered S atoms bonded to Pt1 were refined using a split-atom model (0.75:0.25) with anisotropic displacement parameters (ADPs) restrained to be similar in the two positions (SIMU). The THF molecule was refined with 0.75 occupancy and a common isotropic displacement parameter fixed to 0.12 Å^2^, and restrained to approach 2-fold symmetry with similar C–C distances (SADI). PLATON V-141123 ^92^ software found that the unit cell contains four solvent-accessible voids (175 Å^3^ each) centered at (0, 0, 1/4) and symmetry equivalent positions. These voids host the largest electron density residual (1.14 *e*Å^–3^) and presumably contain disordered and unresolved solvent molecules. Considering the very low *R*-indices reached in the refinement, we made no attempt to model this electron density or to remove its contribution from the data using the SQUEEZE^93^ routine. The unit cell contains 8 PW units and 12 resolvable THF molecules. Assuming that each solvent-accessible void hosts one additional THF molecule, the THF content in the unit cell increases to 16 molecules, and the chemical formula is **2**·2THF, in agreement with analytical evidence. The compound is isomorphous to the published THF solvates of [Ni­(H_2_O)­Pt­(SOCPh)_4_][Bibr ref94] (CCDC 842003) and [Zn­(H_2_O)­Pt­(SOCPh)_4_] (CCDC 1530244).^95^ Their room-temperature crystal structures contain 1.09 and 2 resolvable THF molecules per PW, respectively, and solvent-accessible voids. The crystalline Ni compound before desolvation was analyzed as [Ni­(H_2_O)­Pt­(SOCPh)_4_]·2THF; the Zn compound showed lower THF content and was analyzed as a monotetrahydrofuran solvate. Details on the structure refinement of **2**·0.5tol and **2**·0.75tol are available in the Supporting Information.

PXRD was carried out at room temperature (298 K) with a Malvern-Panalytical Empirean MultiCore X-ray powder diffractometer, equipped with an Empirean tube Cu LFF High Resolution (λ = 1.54056 Å) at 40 kV and 40 mA, and with a capillary spinner sample stage rotating during the measurement (to reduce the impact of preferred orientation of the crystallites). Each sample for PXRD analysis was prepared from freshly crystallized material stored in the mother liquor. Crystals were ground under the solution and filled into a Kapton capillary, which was then sealed with epoxy glue, following the method of Von Dreele.^96^ Data collection was performed in the range 2θ = 3–50° in steps of 0.013°. In Figure 2c, the 2θ-shift of calculated peak positions for **2**·2THF (+0.10° at 2θ = 6.48°; +0.30° at 2θ = 30.1°) is due to low-temperature lattice contraction. The peaks marked with asterisks are due to the Teflon membrane used to pack the sample inside the Kapton capillary. Powder patterns were simulated with Mercury 2025.2.0 ^97^ using a full-width-at-half-maximum of 0.1° in 2θ.

#### 4.1.5 Multifrequency EPR/ENDOR Setup

A superconducting Yttrium Barium Copper Oxide (YBCO) coplanar resonator with a resonant frequency of ∼7.4 GHz was used to deliver the MW pulses addressing the electronic spins, as detailed in refs 36 and 52 . A single crystal of **3**·2THF with a typical size of 0.5 × 0.5 × 0.1 mm was placed at the center of the resonator to maximize its coupling with **B**_MW_, as in Figures 1c and S14b. A cylindrical copper broadband RF coil, terminated with a 50 Ω resistive impedance matching load, was placed on top of the resonator and used to deliver the RF pulses in the 0–150 MHz frequency range. The resonator was kept at 3 K using a Quantum Design Physical Properties Measurement System (QD PPMS), and all experiments reported in this work were carried out at this temperature. Both MW and RF pulses were generated at room temperature and synchronized using different channels of an Arbitrary Waveform Generator. The channel used for RF pulses was first amplified with a 100 W RF power amplifier and then routed toward the RF coil, while the generation of the MW pulses and the detection of the amplitude and phase of the electronic spin echo were performed with a homemade heterodyne setup.^36^

The duration of the MW pulses was calibrated in preliminary experiments by optimizing the echo obtained in a two-MW pulse experiment, as previously reported in refs 36 and 52 . The RF pulses were calibrated using the ENDOR sequence shown in Figure 4a and by determining the RF pulse duration having the strongest effect on the spin echo amplitude (Figure S23).

## Supplementary Material





## Notes

Video of crystal orientation using a polarizing microscope (MP4)
Additional details on synthesis and characterization: crystallographic information and description of other solvates of **2**, IR, UV–vis, and NMR spectra, single-crystal and powder XRD, DC/AC magnetic measurements. Additional details on pulsed EPR measurements: ENDOR measurements and EasySpin simulations, calibration of RF pulses, and sample orientation (PDF)

## References

[1] Manucharyan V. E., Koch J., Glazman L. I., Devoret M. H. (2009). Fluxonium: Single
Cooper-Pair Circuit Free of Charge Offsets. Science.

[2] Nguyen L. B., Goss N., Siva K., Kim Y., Younis E., Qing B., Hashim A., Santiago D. I., Siddiqi I. (2024). Empowering
a Qudit-Based Quantum Processor by Traversing the Dual Bosonic Ladder. Nat. Commun..

[3] Li S., Zhu Y.-K., Zhang W.-X., Pan D.-J., Liao W.-C., Lv Q.-X., Yu H.-F., Yu Y., Xue Z.-Y., Yan H., Zhu S.-L. (2025). Quantum-Enhanced
Parameter Estimation in Multilevel
Systems: Experimental Verification in a Superconducting Qutrit Sensor. Phys. Rev. Lett..

[4] Kristen M., Schneider A., Stehli A., Wolz T., Danilin S., Ku H. S., Long J., Wu X., Lake R., Pappas D. P., Ustinov A. V., Weides M. (2020). Amplitude and Frequency
Sensing of Microwave Fields with a Superconducting Transmon Qudit. npj Quantum Inf..

[5] Morton J. J. L., Bertet P. (2018). Storing Quantum Information
in Spins and High-Sensitivity
ESR. J. Magn. Reson..

[6] Zadrozny J. M., Niklas J., Poluektov O. G., Freedman D. E. (2015). Millisecond Coherence
Time in a Tunable Molecular Electronic Spin Qubit. ACS Cent. Sci..

[7] Atzori M., Morra E., Tesi L., Albino A., Chiesa M., Sorace L., Sessoli R. (2016). Quantum Coherence Times
Enhancement
in Vanadium­(IV)-Based Potential Molecular Qubits: The Key Role of
the Vanadyl Moiety. J. Am. Chem. Soc..

[8] Schäfter D., Wischnat J., Tesi L., De Sousa J. A., Little E., McGuire J., Mas-Torrent M., Rovira C., Veciana J., Tuna F., Crivillers N., van Slageren J. (2023). Molecular
One- and Two-Qubit Systems with Very Long Coherence Times. Adv. Mater..

[9] Chicco S., Chiesa A., Allodi G., Garlatti E., Atzori M., Sorace L., De Renzi R., Sessoli R., Carretta S. (2021). Controlled
Coherent Dynamics of [VO­(TPP)], a Prototype Molecular Nuclear Qudit
with an Electronic Ancilla. Chem. Sci..

[10] Atzori M., Garlatti E., Allodi G., Chicco S., Chiesa A., Albino A., De Renzi R., Salvadori E., Chiesa M., Carretta S., Sorace L. (2021). Radiofrequency
to Microwave
Coherent Manipulation of an Organometallic Electronic Spin Qubit Coupled
to a Nuclear Qudit. Inorg. Chem..

[11] Vasilenko E., Chorakkunnath V. U., Resch J., Jobbitt N., Serrano D., Goldner P., Kuppusamy S. K., Ruben M., Hunger D. (2026). Optically
Detected Nuclear Magnetic Resonance of Coherent Spins in a Molecular
Complex. Nat. Mater..

[12] Goldfarb, D. ; Stoll, S. EPR Spectroscopy: Fundamentals and Methods; John Wiley & Sons, 2018.

[13] DiVincenzo D. P. (2000). The Physical
Implementation of Quantum Computation. Fortschr.
Phys..

[14] Vandersypen L. M. K., Steffen M., Breyta G., Yannoni C. S., Sherwood M. H., Chuang I. L. (2001). Experimental Realization
of Shor’s Quantum Factoring
Algorithm Using Nuclear Magnetic Resonance. Nat..

[15] Vandersypen L. M. K., Chuang I. L. (2005). NMR Techniques for
Quantum Control and Computation. Rev. Mod. Phys..

[16] Thiele S., Vincent R., Holzmann M., Klyatskaya S., Ruben M., Balestro F., Wernsdorfer W. (2013). Electrical
Readout of Individual Nuclear Spin Trajectories in a Single-Molecule
Magnet Spin Transistor. Phys. Rev. Lett..

[17] Godfrin C., Ferhat A., Ballou R., Klyatskaya S., Ruben M., Wernsdorfer W., Balestro F. (2017). Operating Quantum States
in Single Magnetic Molecules: Implementation of Grover’s Quantum
Algorithm. Phys. Rev. Lett..

[18] Chicco S., Allodi G., Chiesa A., Garlatti E., Buch C. D., Santini P., De Renzi R., Piligkos S., Carretta S. (2024). Proof-of-Concept
Quantum Simulator Based on Molecular Spin Qudits. J. Am. Chem. Soc..

[19] Rubín-Osanz M., Bersani L., Chicco S., Allodi G., De Renzi R., Mavromagoulos A., Roy M. D., Piligkos S., Garlatti E., Carretta S. (2026). Implementing the Quantum Fourier Transform on a Molecular
Qudit with Full Refocusing and State Tomography. Nat. Commun..

[20] Yamamoto S., Nakazawa S., Sugisaki K., Sato K., Toyota K., Shiomi D., Takui T. (2015). Adiabatic
Quantum Computing with
Spin Qubits Hosted by Molecules. Phys. Chem.
Chem. Phys..

[21] Sato K., Nakazawa S., Rahimi R., Ise T., Nishida S., Yoshino T., Mori N., Toyota K., Shiomi D., Yakiyama Y., Morita Y., Kitagawa M., Nakasuji K., Nakahara M., Hara H., Carl P., Höfer P., Takui T. (2009). Molecular Electron-Spin Quantum Computers
and Quantum Information
Processing: Pulse-Based Electron Magnetic Resonance Spin Technology
Applied to Matter Spin-Qubits. J. Mater. Chem..

[22] Gurudev
Dutt M. V., Childress L., Jiang L., Togan E., Maze J., Jelezko F., Zibrov A. S., Hemmer P. R., Lukin M. D. (2007). Quantum Register Based on Individual Electronic and
Nuclear Spin Qubits in Diamond. Science.

[23] Mamin H. J., Kim M., Sherwood M. H., Rettner C. T., Ohno K., Awschalom D. D., Rugar D. (2013). Nanoscale Nuclear Magnetic
Resonance with a Nitrogen-Vacancy Spin
Sensor. Sci..

[24] Bradley C. E., Randall J., Abobeih M. H., Berrevoets R. C., Degen M. J., Bakker M. A., Markham M., Twitchen D. J., Taminiau T. H. (2019). A Ten-Qubit Solid-State Spin Register with Quantum
Memory up to One Minute. Phys. Rev. X.

[25] O’Sullivan J., Kennedy O. W., Debnath K., Alexander J., Zollitsch C. W., Šimėnas M., Hashim A., Thomas C. N., Withington S., Siddiqi I., Mølmer K., Morton J. J. L. (2022). Random-Access
Quantum Memory Using Chirped Pulse Phase
Encoding. Phys. Rev. X.

[26] Morton J. J. L., Tyryshkin A. M., Brown R. M., Shankar S., Lovett B. W., Ardavan A., Schenkel T., Haller E. E., Ager J. W., Lyon S. A. (2008). Solid-State Quantum Memory Using
the ^31^P
Nuclear Spin. Nature.

[27] Wolfowicz G., Maier-Flaig H., Marino R., Ferrier A., Vezin H., Morton J. J. L., Goldner P. (2015). Coherent Storage of Microwave Excitations
in Rare-Earth Nuclear Spins. Phys. Rev. Lett..

[28] Brown R. M., Tyryshkin A. M., Porfyrakis K., Gauger E. M., Lovett B. W., Ardavan A., Lyon S. A., Briggs G. A. D., Morton J. J. L. (2011). Coherent
State Transfer between an Electron and Nuclear Spin in ^15^N@C_60_. Phys. Rev. Lett..

[29] Laucht A., Muhonen J. T., Mohiyaddin F. A., Kalra R., Dehollain J. P., Freer S., Hudson F. E., Veldhorst M., Rahman R., Klimeck G., Itoh K. M., Jamieson D. N., McCallum J. C., Dzurak A. S., Morello A. (2015). Electrically
Controlling
Single-Spin Qubits in a Continuous Microwave Field. Sci. Adv..

[30] Lim S., Vaganov M. V., Liu J., Ardavan A. (2025). Demonstrating Experimentally
the Encoding and Dynamics of an Error-Correctable Logical Qubit on
a Hyperfine-Coupled Nuclear Spin Qudit. Phys.
Rev. Lett..

[31] Grezes C., Julsgaard B., Kubo Y., Stern M., Umeda T., Isoya J., Sumiya H., Abe H., Onoda S., Ohshima T., Jacques V., Esteve J., Vion D., Esteve D., Mølmer K., Bertet P. (2014). Multimode Storage and
Retrieval of Microwave Fields in a Spin Ensemble. Phys. Rev. X.

[32] Moreno-Pineda E., Wernsdorfer W. (2025). Magnetic Molecules
as Building Blocks for Quantum Technologies. Adv. Quantum Technol..

[33] Atzori M., Sessoli R. (2019). The Second Quantum
Revolution: Role and Challenges
of Molecular Chemistry. J. Am. Chem. Soc..

[34] Troiani F., Affronte M. (2011). Molecular Spins for
Quantum Information Technologies. Chem. Soc.
Rev..

[35] Troiani F., Ghirri A., Paris M. G. A., Bonizzoni C., Affronte M. (2019). Towards Quantum Sensing with Molecular
Spins. J. Magn. Magn. Mater..

[36] Bonizzoni C., Ghirri A., Santanni F., Affronte M. (2024). Quantum Sensing of
Magnetic Fields with Molecular Spins. npj Quantum
Inf..

[37] Singh H., Souza N. D., Zhong K., Druga E., Oshiro J., Blankenship B., Montis R., Reimer J. A., Breeze J. D., Ajoy A. (2025). Room-Temperature Quantum Sensing
with Photoexcited Triplet Electrons
in Organic Crystals. Phys. Rev. Res..

[38] Shiddiq M., Komijani D., Duan Y., Gaita-Ariño A., Coronado E., Hill S. (2016). Enhancing Coherence
in Molecular
Spin Qubits via Atomic Clock Transitions. Nature.

[39] Atzori M., Tesi L., Morra E., Chiesa M., Sorace L., Sessoli R. (2016). Room-Temperature Quantum
Coherence and Rabi Oscillations
in Vanadyl Phthalocyanine: Toward Multifunctional Molecular Spin Qubits. J. Am. Chem. Soc..

[40] Noh K., Colazzo L., Urdaniz C., Lee J., Krylov D., Devi P., Doll A., Heinrich A. J., Wolf C., Donati F., Bae Y. (2023). Template-Directed 2D Nanopatterning
of *S* = 1/2 Molecular Spins. Nanoscale Horiz..

[41] Choi D. J., Phark S. H., Heinrich A. J., Lorente N. (2025). Electron Spin Resonance
with Scanning Tunneling Microscopy: A Tool for an on-Surface Quantum
Platform of Identical Qubits. Nanoscale Adv..

[42] Sellies L., Spachtholz R., Bleher S., Eckrich J., Scheuerer P., Repp J. (2023). Single-Molecule Electron Spin Resonance by Means of Atomic Force
Microscopy. Nature.

[43] Giaconi N., Tacconi L., Briganti M., Nicolini A., Mironova O., Albanesi M., Lion J., Santanni F., Otero E., Ohresser P., Serrano G., Poggini L., Cornia A., Mannini M. (2026). Deposition of an Addressable
Molecular Spin Qubit with
Built-In Decoupling Structure. J. Am. Chem.
Soc..

[44] Yamauchi A., Yanai N. (2024). Toward Quantum Noses: Quantum Chemosensing Based on Molecular Qubits
in Metal-Organic Frameworks. Acc. Chem. Res..

[45] Yu C. J., von Kugelgen S., Laorenza D. W., Freedman D. E. (2021). A Molecular Approach
to Quantum Sensing. ACS Cent. Sci..

[46] Czap G., Wagner P. J., Xue F., Gu L., Li J., Yao J., Wu R., Ho W. (2019). Probing and
Imaging Spin Interactions
with a Magnetic Single-Molecule Sensor. Science.

[47] Perunicic V. S., Hill C. D., Hall L. T., Hollenberg L. C. L. (2016). A Quantum
Spin-Probe Molecular Microscope. Nat. Commun..

[48] Imperato M., Nicolini A., Borsari M., Briganti M., Chiesa M., Liao Y. K., Ranieri A., Raza A., Salvadori E., Sorace L., Cornia A. (2024). Quantum Spin Coherence and Electron
Spin Distribution Channels in Vanadyl-Containing Lantern Complexes. Inorg. Chem. Front..

[49] Chizzini M., Crippa L., Zaccardi L., Macaluso E., Carretta S., Chiesa A., Santini P. (2022). Quantum Error Correction with Molecular
Spin Qudits. Phys. Chem. Chem. Phys..

[50] Chiesa A., Petiziol F., Macaluso E., Wimberger S., Santini P., Carretta S. (2021). Embedded Quantum-Error Correction
and Controlled-Phase Gate for Molecular Spin Qubits. AIP Adv..

[51] Mezzadri M., Lepori L., Chiesa A., Carretta S. (2025). Dephasing-Tolerant
Quantum Sensing for Transverse Magnetic Fields with Spin Qudits. Quantum Sci. Technol..

[52] Lanza M., Bonizzoni C., Mironova O., Santanni F., Nicolini A., Ghirri A., Cornia A., Affronte M. (2026). Quantum Sensing of
Time-Dependent Magnetic Signals with Molecular Spins. Phys. Rev. Appl..

[53] Bonizzoni C., Ghirri A., Affronte M. (2018). Coherent Coupling of Molecular Spins
with Microwave Photons in Planar Superconducting Resonators. Adv. Phys.: X.

[54] Jenkins M. D., Zueco D., Roubeau O., Aromí G., Majer J., Luis F. (2016). A Scalable Architecture
for Quantum
Computation with Molecular Nanomagnets. Dalton
Trans..

[55] Abhyankar N., Agrawal A., Campbell J., Maly T., Shrestha P., Szalai V. (2022). Recent Advances in
Microresonators and Supporting Instrumentation
for Electron Paramagnetic Resonance Spectroscopy. Rev. Sci. Instrum..

[56] Blank A., Twig Y., Ishay Y. (2017). Recent Trends
in High Spin Sensitivity
Magnetic Resonance. J. Magn. Reson..

[57] Dayan N., Artzi Y., Jbara M., Cristea D., Blank A. (2023). Pulsed Electron-Nuclear
Double Resonance in the Fourier Regime. ChemPhysChem.

[58] Bonizzoni C., Affronte M., Ghirri A., Santanni F., Atzori M., Sorace L., Sessoli R. (2020). Storage and Retrieval
of Microwave
Pulses with Molecular Spin Ensembles. npj Quantum
Inf..

[59] Mims W. B. (1965). Pulsed
Endor Experiments. Proc. R. Soc. London, Ser.
A.

[60] Greenwood, N. N. ; Earnshaw, A. Chemistry of the Elements, 2nd Ed.; Oxford: UK, 1997.

[61] Smith G. D., Caughlan C. N., Campbell J. A. (1972). Crystal and molecular structures
of di-μ-Oxo-bis­(diacetylacetonatotitanium­(IV))-bisdioxane, (TiO­(C_5_H_7_O_2_)_2_)_2_·2C_4_H_8_O_2_, and di-μ-Oxo-bis­(diacetylacetonatotitanium­(IV)),
(TiO­(C_5_H_7_O_2_)_2_)_2_. Inorg. Chem..

[62] Yamabayashi T., Atzori M., Tesi L., Cosquer G., Santanni F., Boulon M. E. E., Morra E., Benci S., Torre R., Chiesa M., Sorace L., Sessoli R., Yamashita M. (2018). Scaling Up
Electronic Spin Qubits into a Three-Dimensional Metal-Organic Framework. J. Am. Chem. Soc..

[63] Fay R. C., Lowry R. N. (1967). Stereochemistry and Lability of Dihalobis­(β-Diketonato)­Titanium­(IV)
Complexes. I. Acetylacetonates. Inorg. Chem..

[64] Wen R., Shen G., Zhai J., Meng L., Bai Y. (2023). Optimization
of TiO­(Acac)_2_ for Efficient Catalytic Alcoholysis of Waste
PET Using Response Surface Methodology. New
J. Chem..

[65] Beach S. A., Guillet J. L., Lagueux S. P., Perfetti M., Livesay B. N., Shores M. P., Bacon J. W., Rheingold A. L., Arnold P. L., Doerrer L. H. (2020). Heterotrimetallic
{LnOVPt} Complexes
with Antiferromagnetic Ln-V Coupling and Magnetic Memory. Chem. Commun..

[66] Mironova O., Bellini G., Nicolini A., Imperato M., Ranieri A., Borsari M., Briganti M., Clérac R., Rouzières M., Salvadori E., Pagliero M. C., Chiesa M., Cornia A. (2026). Oxovanadium (IV) Thiocarboxylate Paddlewheels Containing
Ancillary Group 10 Metals: A Comparative Study on Pd and Pt Derivatives. Inorg. Chem..

[67] Mironova O., Bellini G., Nicolini A., Cornia A. (2025). Multiple Dimerization
Modes in Thiocarboxylate Paddlewheel Complexes: A Comprehensive View
of Energy Landscapes from DFT Calculations and Statistics. Chem. - Eur. J..

[68] Beach S. A., Doerrer L. H. (2018). Heterobimetallic
Lantern Complexes and Their Novel
Structural and Magnetic Properties. Acc. Chem.
Res..

[69] Imperato M., Nicolini A., Boniburini M., Sartini D., Benassi E., Chiesa M., Gigli L., Liao Y.-K., Raza A., Salvadori E., Sorace L., Cornia A. (2024). Dual Structure of a
Vanadyl-Based Molecular Qubit Containing a Bis­(β-Diketonato)
Ligand. Inorg. Chem..

[70] Stoll S., Schweiger A. (2006). EasySpin,
a Comprehensive Software Package for Spectral
Simulation and Analysis in EPR. J. Magn. Reson..

[71] Stewart C. P., Porte A. L. (1972). Electron Paramagnetic
Resonance Spectra of Some Oxovanadium­(IV)
Chelates. J. Chem. Soc., Dalton Trans..

[72] Strach S. J., Bramley R. (1984). EPR of the Vanadyl Ion in Tutton
Salts at Zero Magnetic
Field. Chem. Phys. Lett..

[73] Grant C. V., Ball J. A., Hamstra B. J., Pecoraro V. L., Britt R. D. (1998). ^51^V ESE-ENDOR Studies
of Oxovanadium­(IV) Complexes: Investigation of
the Nuclear Quadrupole Interaction. J. Phys.
Chem. B.

[74] Hahn E. L. (1950). Spin Echoes. Phys. Rev..

[75] Lenz S., Bamberger H., Hallmen P. P., Thiebes Y., Otto S., Heinze K., van Slageren J. (2019). Chromium­(III)-Based Potential Molecular
Quantum Bits with Long Coherence Times. Phys.
Chem. Chem. Phys..

[76] Eaton, S. ; Eaton, G. Distance Measurements in Biological Systems by EPR. In Biological Magnetic Resonance, Vol 19; Springer: New York, NY, 2006; pp 29–129.

[77] Davies E. R. (1974). A New Pulse
Endor Technique. Phys. Lett. A.

[78] Aznar C. P., Deligiannakis Y., Tolis E. J., Kabanos T., Brynda M., Britt R. D. (2004). ESE-ENDOR
Study and DFT Calculations on Oxovanadium
Compounds: Effect of Axial Anionic Ligands on the 51V Nuclear Quadrupolar
Coupling Constant. J. Phys. Chem. A.

[79] Bonizzoni C., Tincani M., Santanni F., Affronte M. (2022). Machine-Learning-Assisted
Manipulation and Readout of Molecular Spin Qubits. Phys. Rev. Appl..

[80] Bonizzoni C., Lamberto D., Napoli S., Günzler S., Rieger D., Santanni F., Ghirri A., Wernsdorfer W., Savasta S., Affronte M. (2026). Observation of Perfect
Absorption
in Hyperfine Levels of Molecular Spins with Hermitian Subspaces. Nat. Commun..

[81] Lenz S., König D., Hunger D., van Slageren J. (2021). Room-Temperature
Quantum Memories Based on Molecular Electron Spin Ensembles. Adv. Mater..

[82] Simas A. B. C., Pereira V. L. P., Barreto C. B., de Sales D. L., de Carvalho L. L. (2009). An Expeditious
and Consistent Procedure
for Tetrahydrofuran (THF) Drying and Deoxygenation by the Still Apparatus. Quim. Nova.

[83] Fulmer G. R., Miller A. J. M., Sherden N. H., Gottlieb H. E., Nudelman A., Stoltz B. M., Bercaw J. E., Goldberg K. I. (2010). NMR Chemical Shifts
of Trace Impurities: Common Laboratory Solvents, Organics, and Gases
in Deuterated Solvents Relevant to the Organometallic Chemist. Organometallics.

[84] Wu D. H., Chen A., Johnson C. S. (1995). An Improved Diffusion-Ordered Spectroscopy
Experiment Incorporating Bipolar-Gradient Pulses. J. Magn. Reson., Ser. A.

[85] Bachmann S., Neufeld R., Dzemski M., Stalke D. (2016). New External Calibration
Curves (ECCs) for the Estimation of Molecular Weights in Various Common
NMR Solvents. Chem. - Eur. J..

[86] Crockett M. P., Zhang H., Thomas C. M., Byers J. A. (2019). Adding Diffusion
Ordered NMR Spectroscopy (DOSY) to the Arsenal for Characterizing
Paramagnetic Complexes. Chem. Commun..

[87] Bachmann S., Gernert B., Stalke D. (2016). Solution Structures
of Alkali Metal
Cyclopentadienides in THF Estimated by ECC-DOSY NMR-Spectroscopy (Incl.
Software). Chem. Commun..

[88] APEX2, SADABS, SAINT; Bruker-AXS; Inc.: Madison, Wisconsin, USA, 2012.

[89] Farrugia L.
J. (2012). WinGX and
ORTEP for Windows: An Update. J. Appl. Crystallogr..

[90] Altomare A., Cascarano G., Giacovazzo C., Guagliardi A., Burla M. C. C., Polidori G., Camalli M. (1994). SIR 92 – a Program
for Automatic Solution of Crystal Structures by Direct Methods. J. Appl. Crystallogr..

[91] Sheldrick G. M. (2015). Crystal
Structure Refinement with SHELXL. Acta Crystallogr.,
Sect. C: Struct. Chem..

[92] Spek A. L. (2003). Single-Crystal
Structure Validation with the Program PLATON. J. Appl. Crystallogr..

[93] Spek A. L. (2015). PLATON
SQUEEZE: A Tool for the Calculation of the Disordered Solvent Contribution
to the Calculated Structure Factors. Acta Crystallogr.,
Sect. C: Struct. Chem..

[94] Dahl E. W., Baddour F. G., Fiedler S. R., Hoffert W. A., Shores M. P., Yee G. T., Djukic J. P., Bacon J. W., Rheingold A. L., Doerrer L. H. (2012). Antiferromagnetic
Coupling across a Tetrametallic Unit
through Noncovalent Interactions. Chem. Sci..

[95] Baddour F. G., Hyre A. S., Guillet J. L., Pascual D., Lopez-De-Luzuriaga J.
M., Alam T. M., Bacon J. W., Doerrer L. H. (2017). Pt-Mg, Pt-Ca, and
Pt-Zn Lantern Complexes and Metal-Only Donor-Acceptor Interactions. Inorg. Chem..

[96] Von
Dreele R. B. (2006). A Rapidly Filled Capillary Mount for Both Dry Powder
and Polycrystalline Slurry Samples. J. Appl.
Crystallogr..

[97] Macrae C. F., Sovago I., Cottrell S. J., Galek P. T. A., McCabe P., Pidcock E., Platings M., Shields G. P., Stevens J. S., Towler M., Wood P. A. (2020). Mercury
4.0: From Visualization to
Analysis, Design and Prediction. J. Appl. Crystallogr..

