# Supplementary material for: Storage and Retrieval of Electronic Spin Coherence Using Nuclear Spins in a Vanadyl Paddlewheel
Source: J Am Chem Soc. 2026 Aug 10;148(39):42079–90. doi: 10.1021/jacs.6c10524 (PMC13651529; doi:10.1021/jacs.6c10524)
Supplement: Supplementary file 1 [file ja6c10524_si_002.pdf]

Supporting information

Storage and retrieval of electronic spin coherence using nuclear spins in a vanadyl paddlewheel

Matteo Lanza,[a,b∇]* Olga Mironova,[c∇]* Claudio Bonizzoni,[a,b]* Giacomo Bellini,[c] Alessio Nicolini,[c] Alberto Ghirri,[b] Rodolphe Clérac,[d] Mathieu Rouzières,[d] Andrea Cornia,[c]* and Marco Affronte[a,b]

Table of contents

Synthesis and characterization 2

X-ray structure refinement of **2**·0.5tol and **2**·0.75tol 2
Supplementary note 1: additional synthetic and structural details 2
NMR, IR, and UV-Vis spectroscopy 4
Supplementary note 2: ^{1}H DOSY NMR 7
X-ray diffraction 9

Direct-current (DC) and alternating-current (AC) magnetic measurements 10

EPR and ENDOR details 14

Crystal sample orientation 14
Supplementary note 3: EasySpin simulation of the CW-EPR 14
^{1}H ENDOR signal 15
Additional ^{51}V Mims-ENDOR spectra 16
Supplementary note 4: ^{195}Pt contribution to Mims-ENDOR spectra 17
Comparison between Davies-ENDOR and Mims-ENDOR 19
Test of storage block efficiency 20
Tests of the full SR protocol 20
Supplementary note 5: Estimation of *in-plane fidelity* of the SR protocol 22
Calibration of the duration of the RF pulses 23

References 24

[∇]M.L. and O.M. contributed equally. *E-mail: matteo.lanza@unimore.it, mironovaoa.nsk@gmail.com, claudio.bonizzoni@unimore.it, acornia@unimore.it

[a]Dipartimento di Scienze Fisiche, Informatiche e Matematiche, Università degli Studi di Modena e Reggio Emilia, via G. Campi 213/A, 41125 Modena, Italy.

[b]Istituto Nanoscienze – CNR, S3, via G. Campi 213/A, 41125 Modena, Italy

[c]Dipartimento di Scienze Chimiche e Geologiche e UdR INSTM, Università degli Studi di Modena e Reggio Emilia, via G. Campi 103, 41125 Modena, Italy.

[d]Univ. Bordeaux, CNRS, CRPP, UMR 5031, F-33600 Pessac, France.

Synthesis and characterization

X-ray structure refinement of 2·0.5tol and 2·0.75tol

In **2**·0.5tol, the toluene molecule is orientationally disordered around a twofold axis. It was refined, treating the Ph ring as a rigid hexagon (AFIX 66) and applying geometric restraints to enforce approximate *mm*2 symmetry. To this end, the methyl C atom was restrained to be coplanar with the phenyl ring (FLAT) and at equal distances from the ortho C atoms (SADI). In addition, the C–CH_3 distance was restrained to 1.51(2) Å (DFIX). The toluene C atoms were treated isotropically with U values restrained to be similar (SIMU).

In **2**·0.75tol, one full-occupancy toluene molecule in general position was clearly located and refined with similar restraints on aromatic C–C bond distances (SADI) and rigid-bond restraints on the ADPs of C atoms (RIGU). The unit cell contains two solvent-accessible voids (317 $Å^3$ each) centered at the symmetry-equivalent sites (0, 1/2, 1) and (1/2, 1, 1/2). These voids host the largest electron density residual (1.55 eÅ^{-3}) and presumably contain disordered and unresolved solvent molecules. We removed their contribution from the data using the SQUEEZE[1] routine in PLATON V-141123,[2] which significantly reduced $R1[I > 2\sigma(I)]$ from 4.05 to 3.47% and $wR2$(all) from 12.40 to 8.08%. Each void was assumed to host one additional toluene molecule, yielding 6 toluene molecules per unit cell, i.e., 0.75 toluene molecules per paddlewheel unit.

Supplementary note 1: additional synthetic and structural details

A major difficulty encountered during the purification of **2** by crystallization is its tendency to precipitate upon contact with a "bad" solvent like *n*-hexane or diethyl ether. Initial attempts led to the discovery of other solvates, such as **2**·1.5DCM (obtained as long plate-shaped crystals by slow evaporation of *n*-hexane/DCM solution). According to preliminary structural data, this solvate also consists of staggered dimers.

Upon recrystallization from toluene, [VOPt(SOCPh)$_4$] (**1**) forms three solvatomorphic modifications: **1**·0.875tol ($P\overline{1}$), α-**1**·0.5tol ($C2/c$), and β-**1**·0.5tol ($P\overline{1}$) (main structural parameters summarized in **Table S1**).[3] α- and β-**1**·0.5tol contain staggered and bent dimers, respectively, while both types of dimers are present in **1**·0.875tol. Crystallized in the same way, **2** also yields two toluene solvates. The minority phase, **2**·0.5tol ($C2/c$), is isomorphic to α-**1**·0.5tol and displays no significant structural differences, besides showing no resolvable disorder in the PtS_4 units. The main phase, **2**·0.75tol ($P2_1/n$), is structurally similar (although not isomorphic) to **1**·0.875tol, with a comparable unit cell volume and Z = 8. Due to the more symmetric space group, the

asymmetric unit contains one instead of two molecular pairs, but crystal packing is otherwise similar, with π-stacking interactions between Ph substituents (**Figure S1a**). The geometrical parameters of the dimers (ϕ, γ angles, Pt···Pt' and Pt···S' distances) are close to those found in mol3···mol4 of **1**·0.875tol (**Figure S1b**). In particular, the value of the γ angle between the Ti–Pt axes (9.8°) classifies the dimers as *bent*.[3] The arrangement of dimers within the unit cell of **2**·0.75tol is not the most favorable for EPR experiments on a single crystal. The pronouncedly bent configuration of the dimers and their relative orientation create four inequivalent V–Pt vectors at angles of 9.8, 41.8, 51.0, and 59.8°, which would significantly complicate the interpretation of the spectra. For this reason, tetragonal **2**·2THF was preferred.

Table S1. Selected bond distances and angles in solvatomorphs of **2** and **1**.

		Distances (Å)[a]				Angles (°)[a]			
Compound		Tr=O	Tr–Pt	Pt···Pt'	Pt···S' [b]	Tr–Pt···Pt' (ϕ)	τ_{intra}[c]	τ_{inter}[d]	γ[e]
**2**·2THF		1.631(4)	2.8613(10)	3.0814(4)	3.84-3.84	180	13.4[f]	41.1[f]	0
**2**·0.5tol (*C*2/*c*)		1.615(3)	2.8942(8)	3.2406(7)	3.8932(16)	176.10(3)	1.8	–	7.42(3)
**2**·0.75tol (*P*$\bar{1}$)	mol1	1.608(3)	2.8813(8)	3.2163(5)	3.8531(15)	171.221(18)	10.8	–	9.79(2)
	mol2	1.615(3)	2.8623(8)		3.7101(14)	175.637(17)	13.8		
**1**·0.875tol (*P*$\bar{1}$)	mol1	1.582(5)	2.8582(14)	3.1744(5)	3.848(2)	176.62(3)	11.6	42.2	1.96(5)
	mol2	1.577(5)	2.8256(14)		3.827(2)	176.12(3)	16.1		
	mol3	1.566(5)	2.8389(14)	3.2342(5)	3.84-3.87	170.35(3)	–	–	11.87(4)
	mol4	1.575(5)	2.8256(13)		3.74-3.74	175.06(3)	–		
α-**1**·0.5tol (*C*2/*c*)		1.581(2)	2.8321(5)	3.1740(2)	3.84-3.88	176.381(11)	–	–	6.890(16)
β-**1**·0.5tol (*P*$\bar{1}$)	mol1	1.586(2)	2.7997(5)	4.5100(2)	3.3761(9)	135.41(1)	17.0	–	19.311(15)
	mol2	1.581(2)	2.8138(5)		3.3426(8)	126.84(1)	16.8		

[a]Dots indicate intermolecular contacts; primed symbols are used for atoms of the neighboring PW in a dimer. [b]Shortest Pt···S' contact; a range is reported for PtS_4 moieties showing resolvable rotational disorder. [c]Intramolecular twisting angle, evaluated as the average O–Tr–Pt–S dihedral angle (with O and S belonging to the same thiocarboxylate ligand); unless otherwise noted, it is calculated only for PtS_4 moieties not affected by resolvable rotational disorder. [d]Intermolecular twisting angle for coaxial or quasi-coaxial dimers ($\phi \sim 180°$, $\gamma \leq 5°$), evaluated by averaging the smallest set of S–Pt···Pt'–S' dihedrals. [e]Smallest angle between the two Tr–Pt axes. [f]For the 0.75-occupancy component of the rotationally disordered PtS_4 moiety.

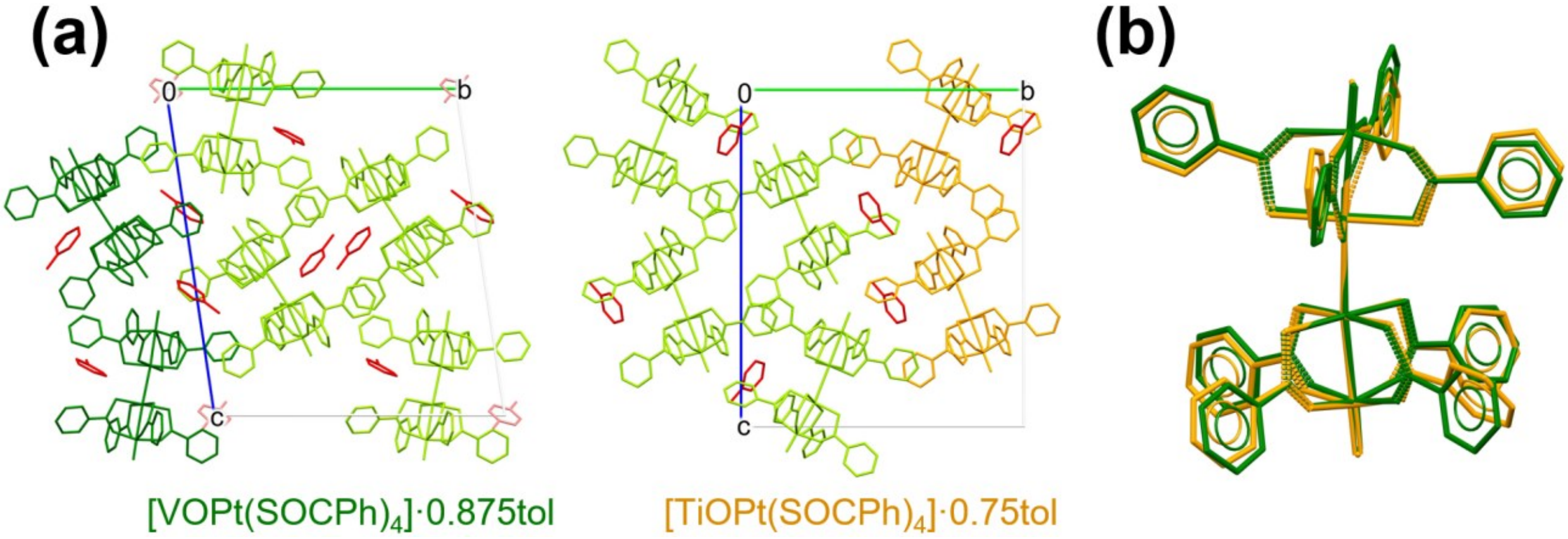

Figure S1. *(a)* 1.5 unit cells of **1**·0.875tol and **2**·0.75tol with toluene molecules colored in red (fully occupied) and pink (partially occupied), similar parts of packing colored in bright green. Capped stick model, hydrogen atoms omitted. *(b)* Overlay of the asymmetric unit of **2**·0.75tol (yellow) and mol3···mol4 in **1**·0.875tol (green).

NMR, IR, and UV-Vis spectroscopy

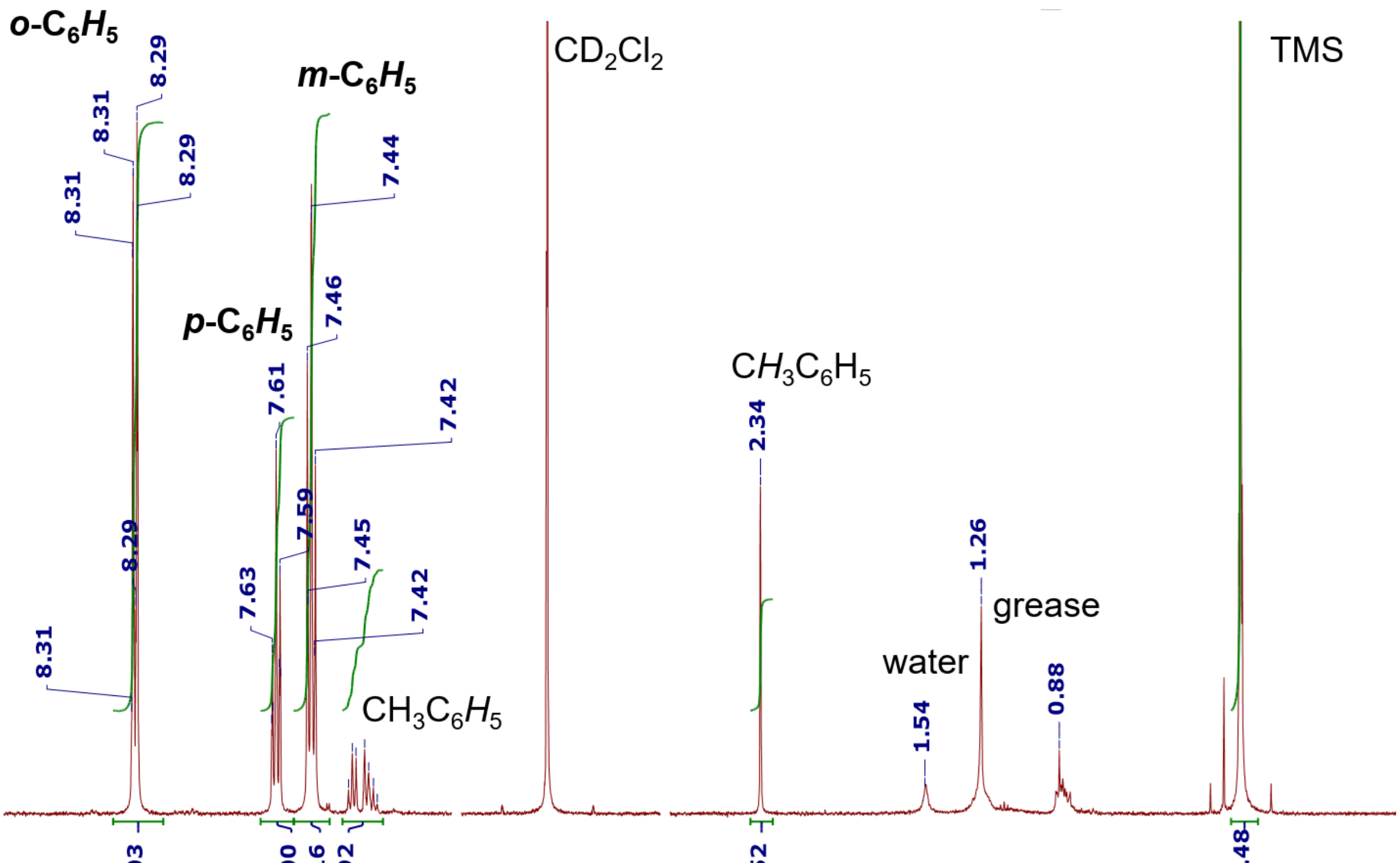

Figure S2. ^{1}H spectrum of **2** in CD_2Cl_2. Toluene remained after crystallization. TMS was added as an internal standard.

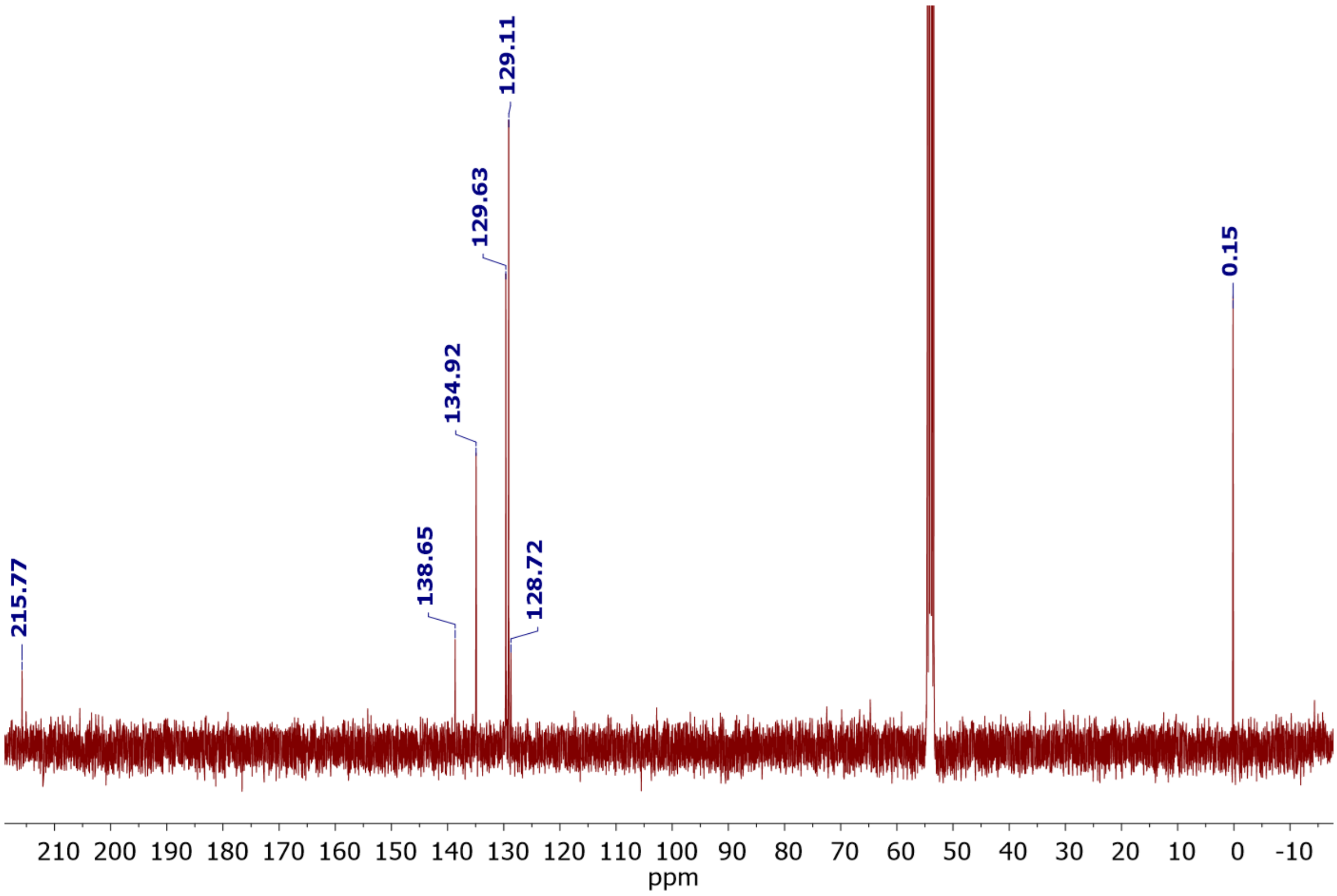

Figure S3. ^{13}C spectrum of **2** in CD_2Cl_2. Toluene remained after crystallization. TMS was added as an internal standard.

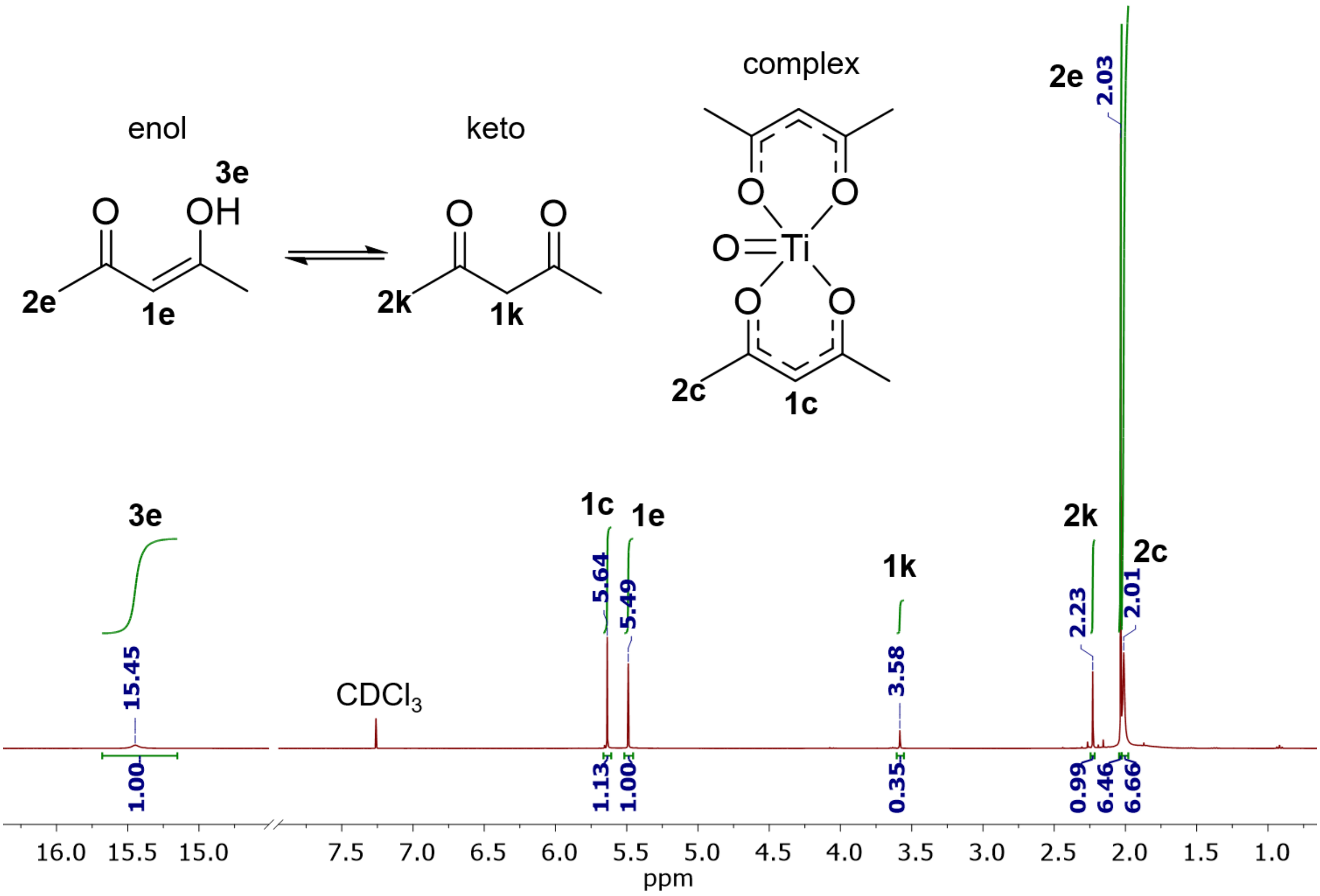

Figure S4. ^{1}H NMR spectrum of $TiO(acac)_2$ (TCI, 99%) in $CDCl_3$.

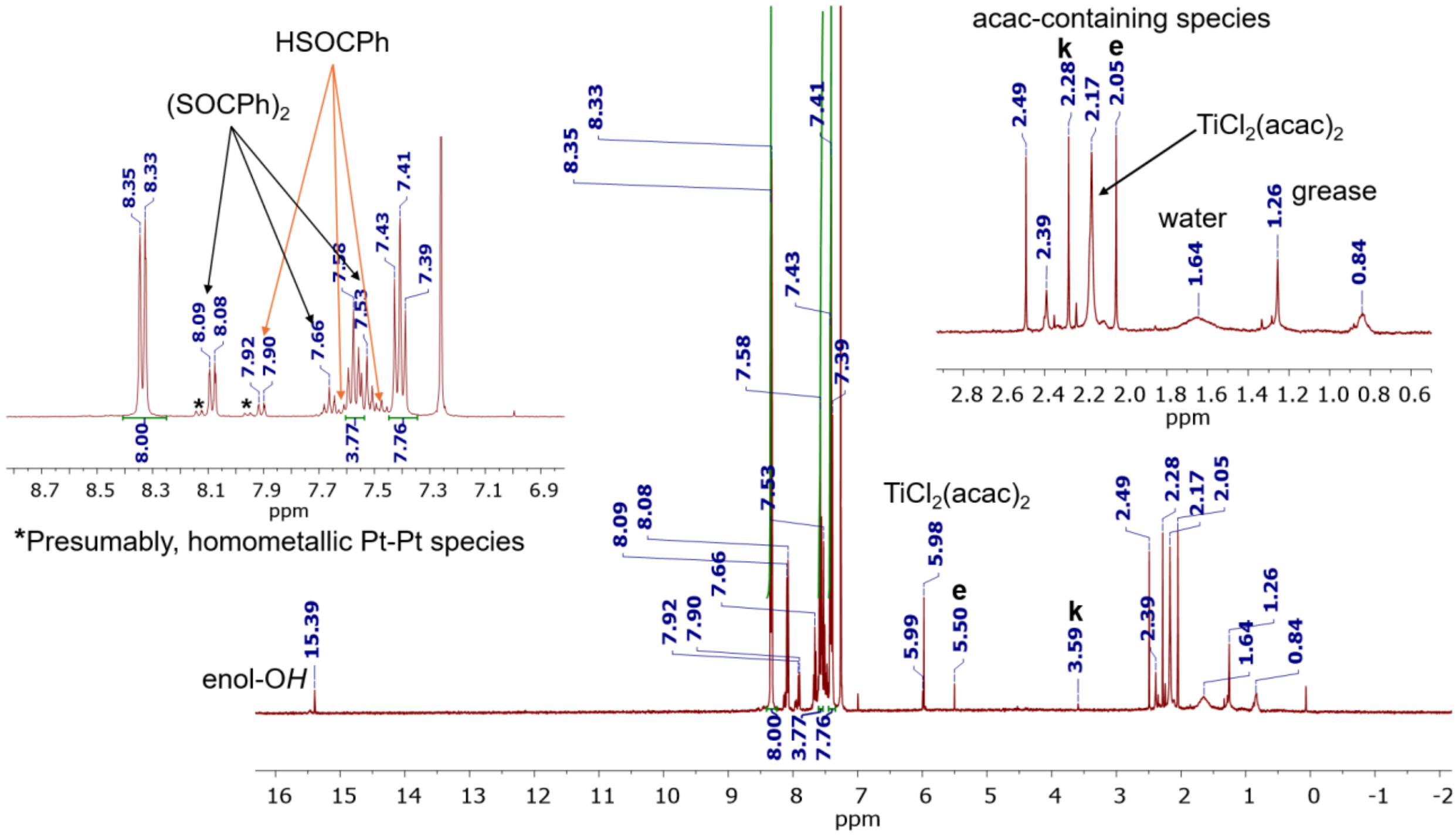

Figure S5. ^{1}H NMR spectrum of the raw **2** in $CDCl_3$. **k** denotes the diketo-form of acetylacetone, **e** the keto-enol.

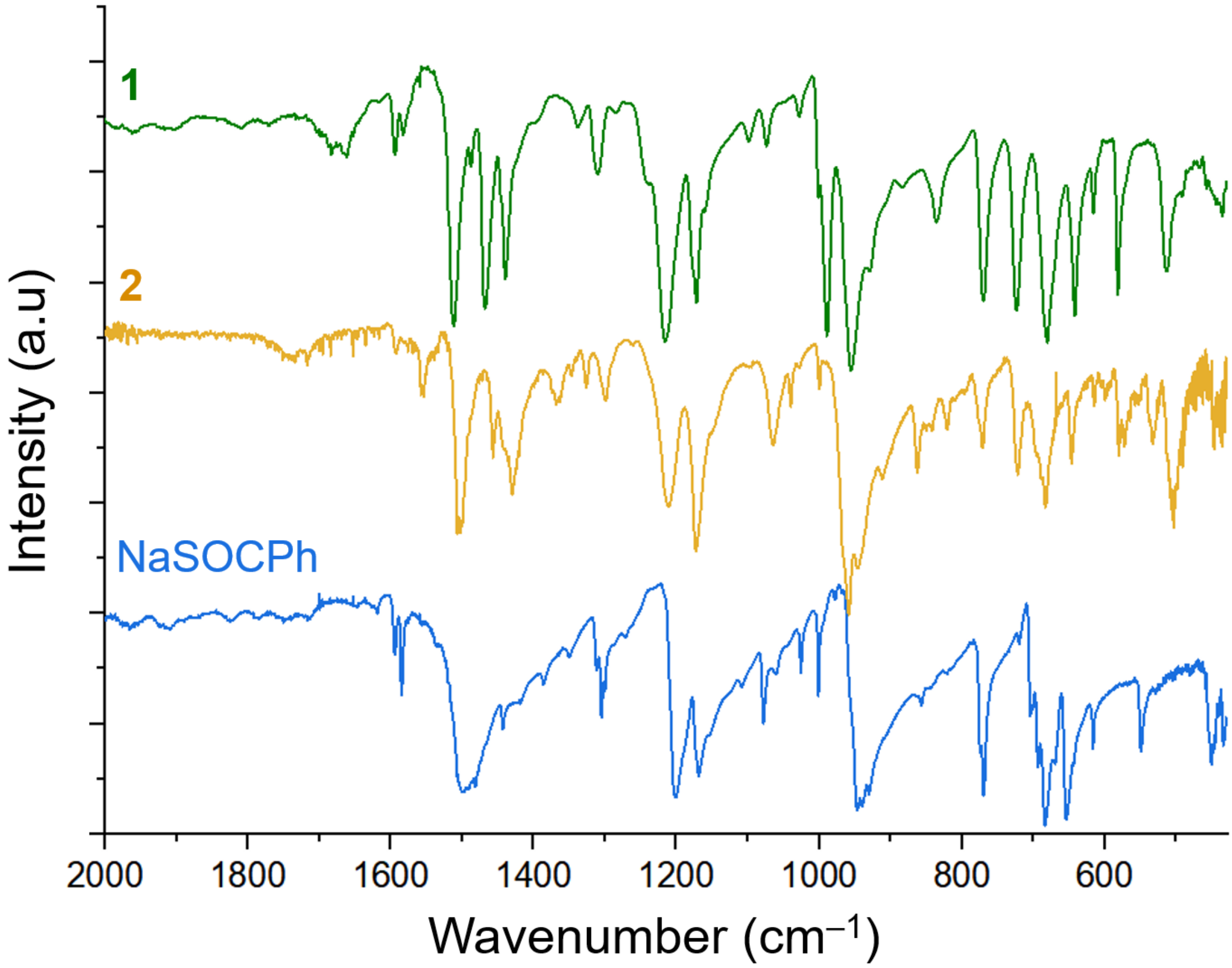

Figure S6. IR spectra of **1**, **2**, and NaSOCPh.

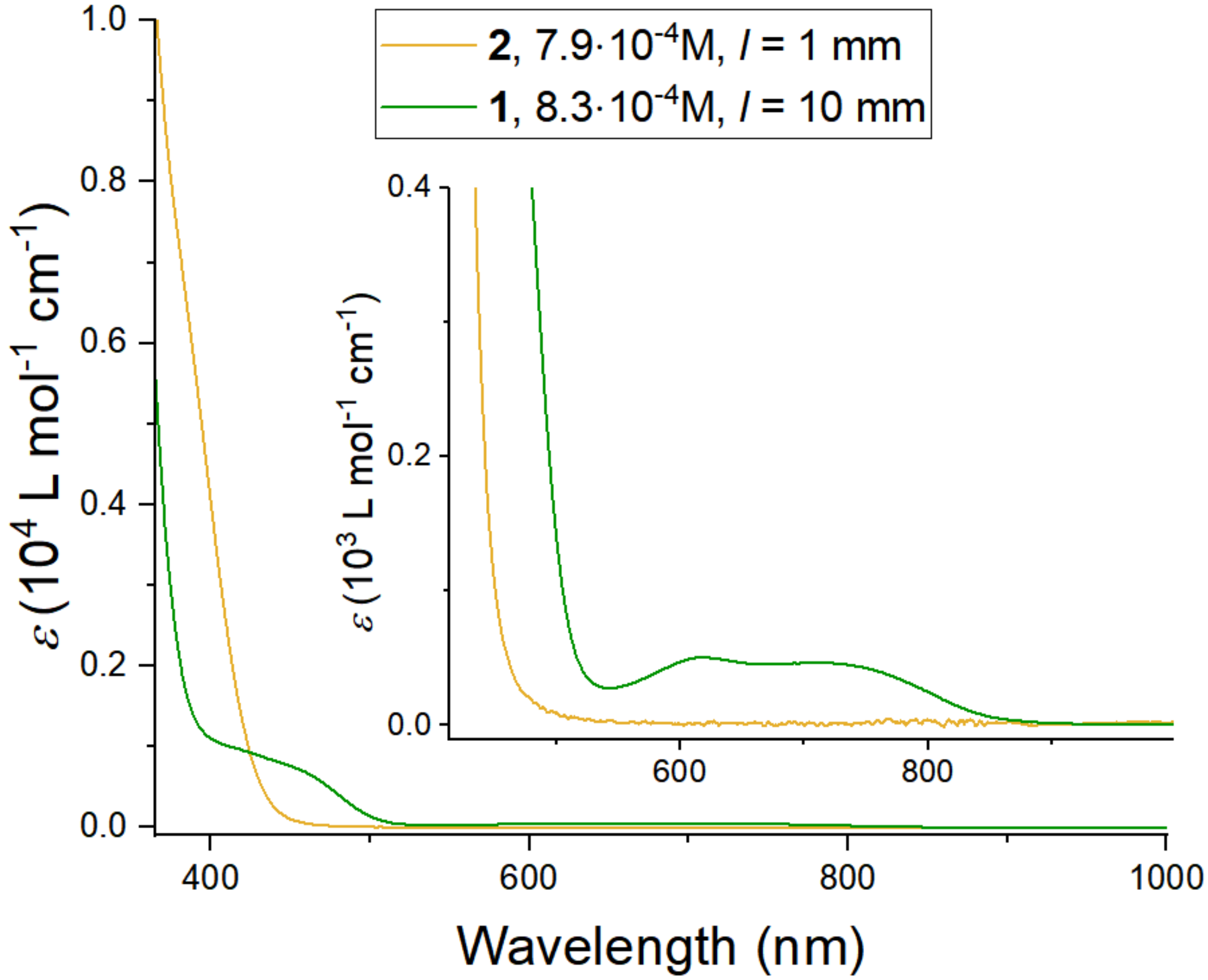

Figure S7. Electronic absorption spectra of **1** and **2** in DCM.[4]

Supplementary note 2: ^{1}H DOSY NMR

The diffusion coefficient (*D*) of **2** ($9.5\cdot10^{-10}$ m^2 s^{-1}) was measured through ^{1}H DOSY spectroscopy (**Figure S8**) and is comparable with that reported for **1** ($D = 9.0\cdot10^{-10}$ m^2 s^{-1}).[4] The value of *D* can be used to directly estimate the molecular weight (*MW*) of the complex using external calibration curves (ECCs).[5,6] Depending on the model, *MW* was estimated as 511±152 (ECC "dissipated spheres and ellipsoids") and 566±183 g mol^{-1} (ECC "merge"). ECC reported by Byers et al.,[7] accounting for the presence of 3d transition elements, afforded *MW* = 573±252 g mol^{-1}. The systematic underestimation of *MW* by ^{1}H DOSY is thus mainly due to the presence of the heavy Pt atom, as already reported.[4] In conclusion, the estimated *MW* and *D* calculated by ^{1}H DOSY strongly indicate that **2** is present in monomeric form (*MW* = 807.66 g mol^{-1}) in CD_2Cl_2, complementing the investigation on paramagnetic **1**[4] and [$VOPd(SOCPh)_4$].[8]

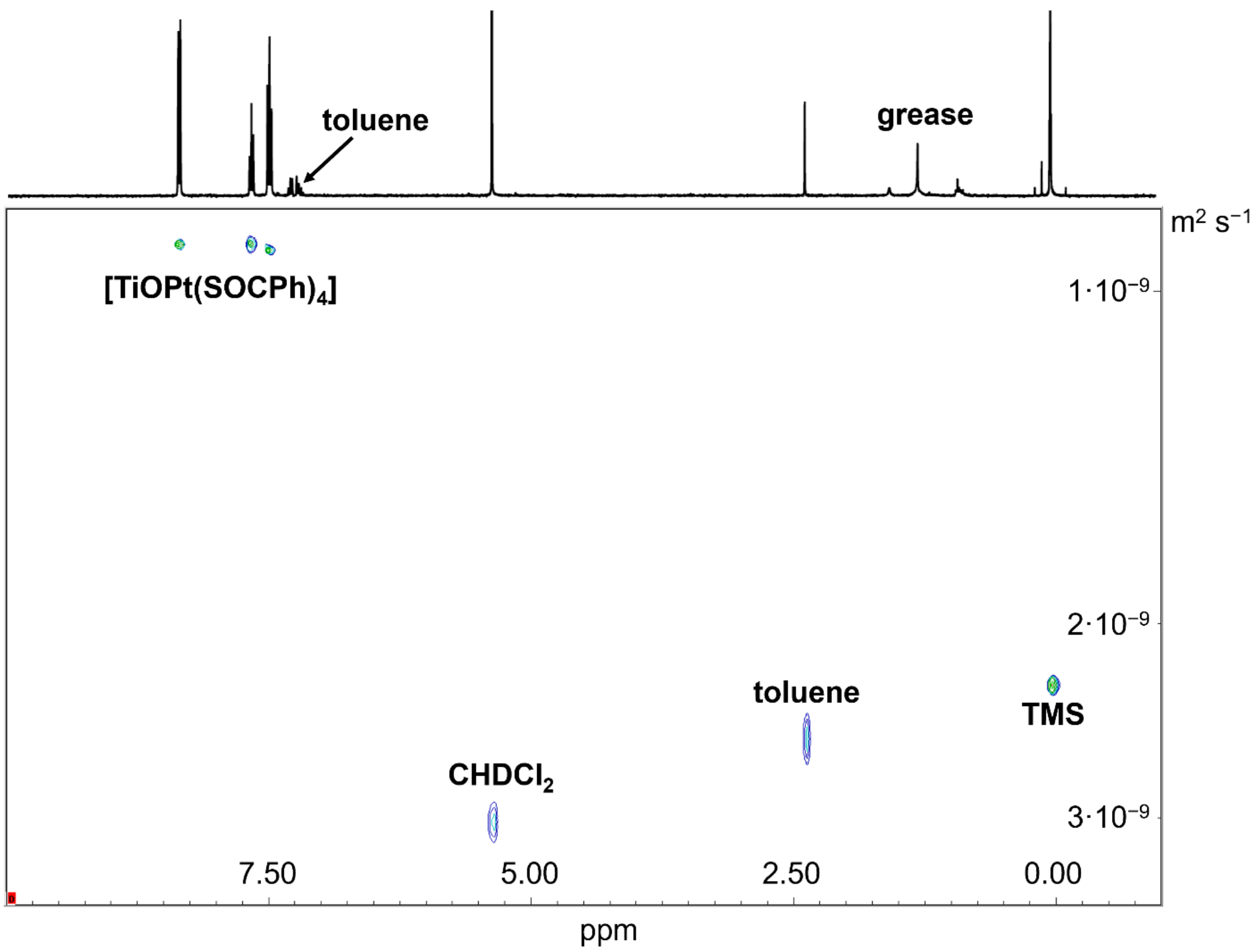

Figure S8. ^{1}H DOSY NMR spectrum of **2** in CD_2Cl_2 (0.006 M, 298 K, 400.13 MHz).

X-ray diffraction

Table S2. Crystal data and refinement parameters for [TiOPt(SOCPh)$_4$] solvatomorphs.

	**2**·2THF	**2**·0.5tol	**2**·0.75tol
CCDC numbers	2477890	2547862	2547863
Radiation	Mo-Kα (λ = 0.71073 Å)	Mo-Kα (λ = 0.71073 Å)	Mo-Kα (λ = 0.71073 Å)
Chemical formula	$C_{36}H_{36}O_7S_4TiPt$	$C_{31.5}H_{24}O_5S_4TiPt$	$C_{33.25}H_{26}O_5S_4TiPt$
Formula weight	951.88	853.74	876.77
T (K)	200(2)	298(2)	298(2)
Crystal size (mm^3)	0.21 × 0.21 × 0.11	0.16 × 0.10 × 0.03	0.12 × 0.06 × 0.01
Crystal system	tetragonal	monoclinic	monoclinic
Space group	$I\bar{4}c2$	$C2/c$	$P2_1/n$
a (Å)	18.9732(3)	15.5168(3)	11.1195(16)
b (Å)	18.9732(3)	16.4928(4)	22.535(4)
c (Å)	22.3812(6)	26.1595(5)	27.209(4)
α (deg)	90	90	90
β (deg)	90	98.0699(10)	99.707(6)
γ (deg)	90	90	90
V (Å^3)	8056.8(3)	6628.3(2)	6720.1(17)
Z	8	8	8
ρ_{calcd} (g cm^{-3})	1.569	1.711	1.733
$2\vartheta_{min}/2\vartheta_{max}$ (deg)	7.082/58.008	6.932/57.088	6.812/53.298
Reflections collected/independent	46117/5359	34043/8336	61739/14055
№ of parameters/ restraints	211/17	368/13	766/60
$R1/wR2$ (all data)	0.0299/0.0851	0.0822/0.0951	0.0662/0.0808
$R1/wR2$ ($I > 2\sigma(I)$)	0.0252/0.0810	0.0392/0.0819	0.0347/0.0730
GOF	1.138	1.018	1.022
Largest diff. peak/hole (eÅ^{-3})	1.14/-0.58	0.87/-0.95	1.13/-0.91

Direct-current (DC) and alternating-current (AC) magnetic measurements

Magnetic susceptibility measurements were performed on a Quantum Design SQUID MPMS-XL magnetometer housed at the Centre de Recherche Paul Pascal (Bordeaux, France). DC magnetic measurements were carried out on a polycrystalline sample of **4**·2THF (31.2 mg) with a nominal doping level x = 0.10 (0.090(2) mol % of V according to ICP-MS). The sample was placed in a sealed double polyethylene/polypropylene bag (3×0.5×0.02 cm; 29.5 mg) in a glovebox with a controlled argon atmosphere. Prior to the experiments, the field-dependent magnetization was measured at 100 K in order to detect the presence of any bulk ferromagnetic impurities. As expected for paramagnetic or diamagnetic materials, a perfectly linear dependence of the magnetization that extrapolates to zero at zero DC field was observed.

Magnetization (M) vs. temperature (T) data of **4**·2THF were collected from 1.85 to 50 K at an applied static field H = 10 kOe and from 1.85 to 38 K at H = 1 kOe. Additionally, the isothermal magnetization was measured as a function of applied field for H = 0÷70 kOe at 1.85 K. The data were reduced using MW = 952 g mol^{-1} and corrected for the sample holder and the intrinsic diamagnetism of the compound (–476·10^{-6} emu mol^{-1}). Magnetic susceptibility (χ) was evaluated as M/H. At 1 kOe, the $\chi_{M}T$ vs. T product, where χ_{M} is the molar magnetic susceptibility, conforms to simple Curie behavior over the whole explored temperature range, indicating isolated spins. Taking g = 1.97 and S =1/2 for vanadium(IV), the average $\chi_{M}T$ = 0.03334(9) cm^3 K mol^{-1} is consistent with x = 0.0916. M vs. H data reach 0.093 μ_B at the highest measured field and are accurately reproduced by a simple Brillouin function behavior with isotropic g fixed at 1.97 and x = 0.09350(8), as pictured in **Figure S9**. The actual vanadium(IV) content is thus consistent with that found by ICP-MS.

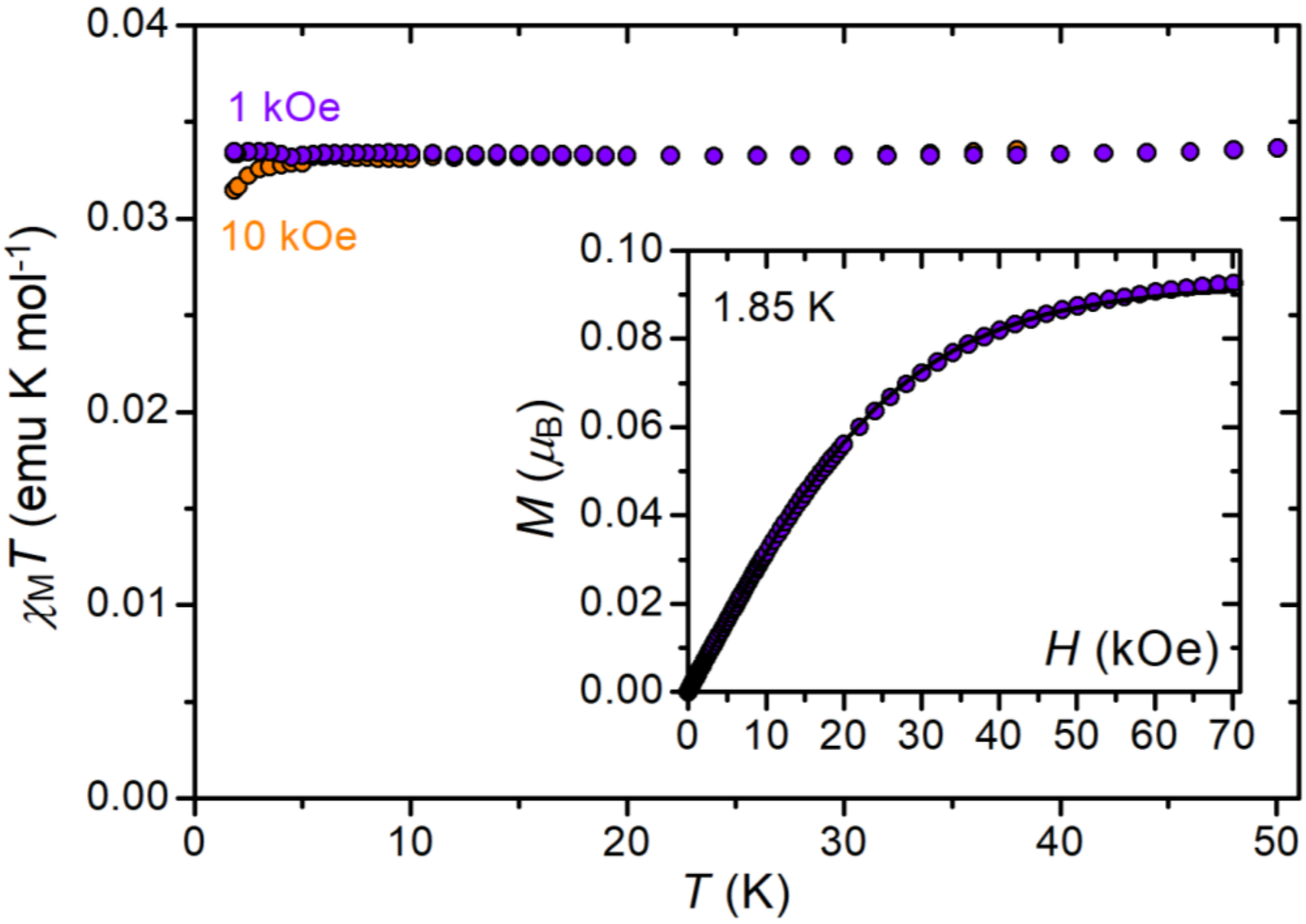

Figure S9. $\chi_M T$ vs. T measured at 1 kOe (violet dots) and 10 kOe (orange dots) on a polycrystalline sample of **4**·2THF with nominal doping level $x = 0.10$. The inset shows M vs. H data recorded at 1.85 K and the best-fit curve based on simple Brillouin function behavior (solid black trace).

The AC magnetic susceptibility measurements were performed in an oscillating AC field of 5 Oe with frequencies between 1 and 1500 Hz. All AC data processing and the final modeling of the relaxation processes were performed using the pyMagSuite software.[9] The molar AC susceptibility data (χ' and χ'') measured as a function of ν, T, and H are provided in **Figures S10–S13** together with the generalized Debye model used to extract the τ values. The field dependence of the spin lattice relaxation time (τ) was estimated at 1.85 and 3.2 K with H up to 10 and 4 kOe, respectively. The temperature dependence of τ was also measured from 1.85 to 10 K at a constant static field of 3.5 kOe.

The relaxation rate was modeled according to:

$$\tau^{-1} = C\frac{1+C_1H^2}{1+C_2H^2}T^n + \frac{B_1}{1+B_2H^2} \qquad \text{(S1)}$$

which includes Raman and quantum tunneling contributions to the magnetization relaxation, respectively. As show in **Figure S13**, the $\tau(T, H)$ data are very well reproduced by the **eq S1** with a single set of parameters: C = 80(20) K^{-n}s^{-1}, C_1 = 2.5(5) 10^2 T^{-2}, C_2 = 1.9(5)·10^4 T^{-2}, n = 2.9(2), B_1 = 1.6(4) 10^2 s^{-1}, B_2 = 60(20) T^{-2}.

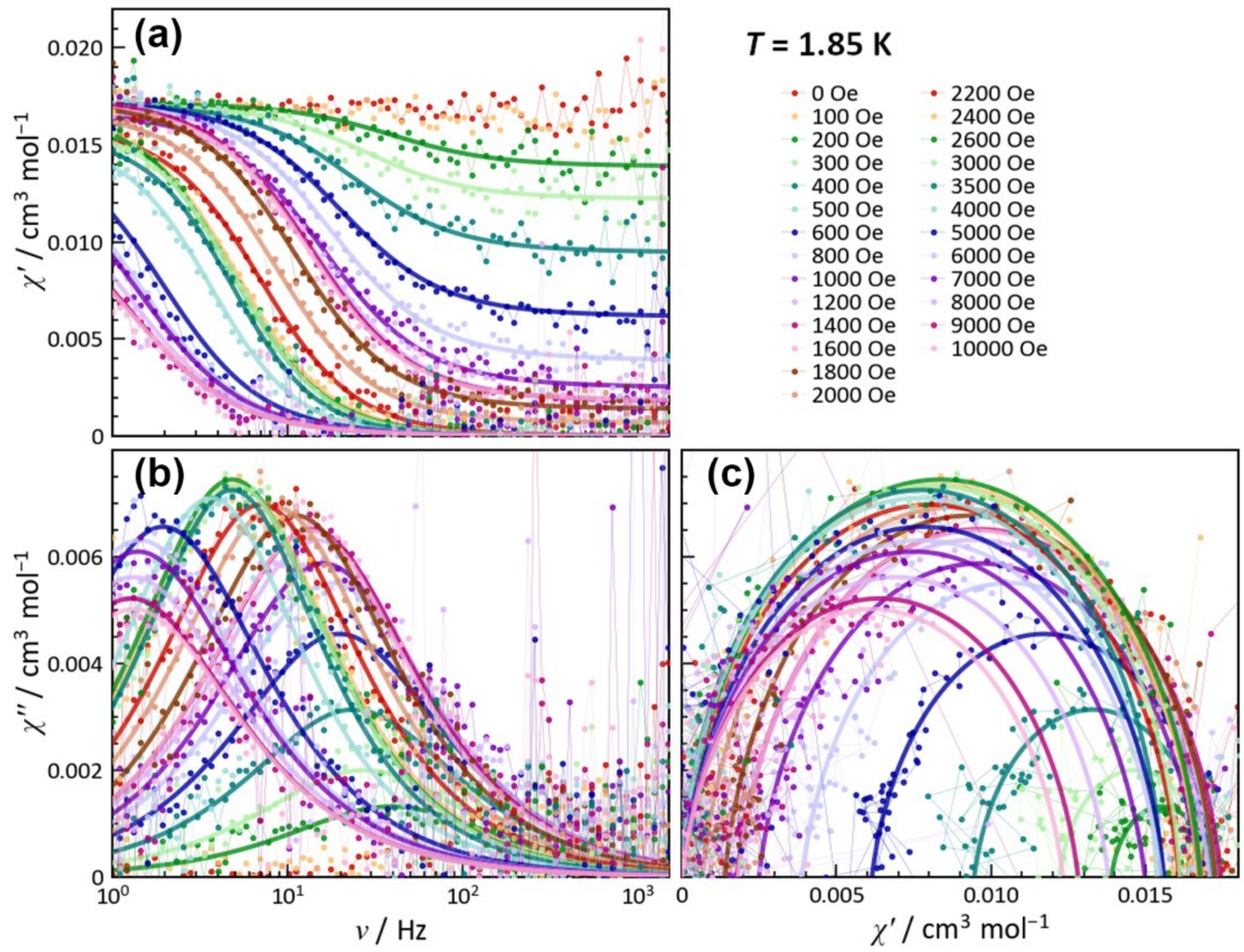

Figure S10. AC susceptibility data plotted as (a) χ' vs. ν, (b) χ'' vs. ν, and (c) χ'' vs. χ' (Cole-Cole plot) measured at 1.85 K under *H* between 0 and 10 kOe on the same sample as in **Figure S9**, and their best-fit curves using the generalized Debye model (solid traces).

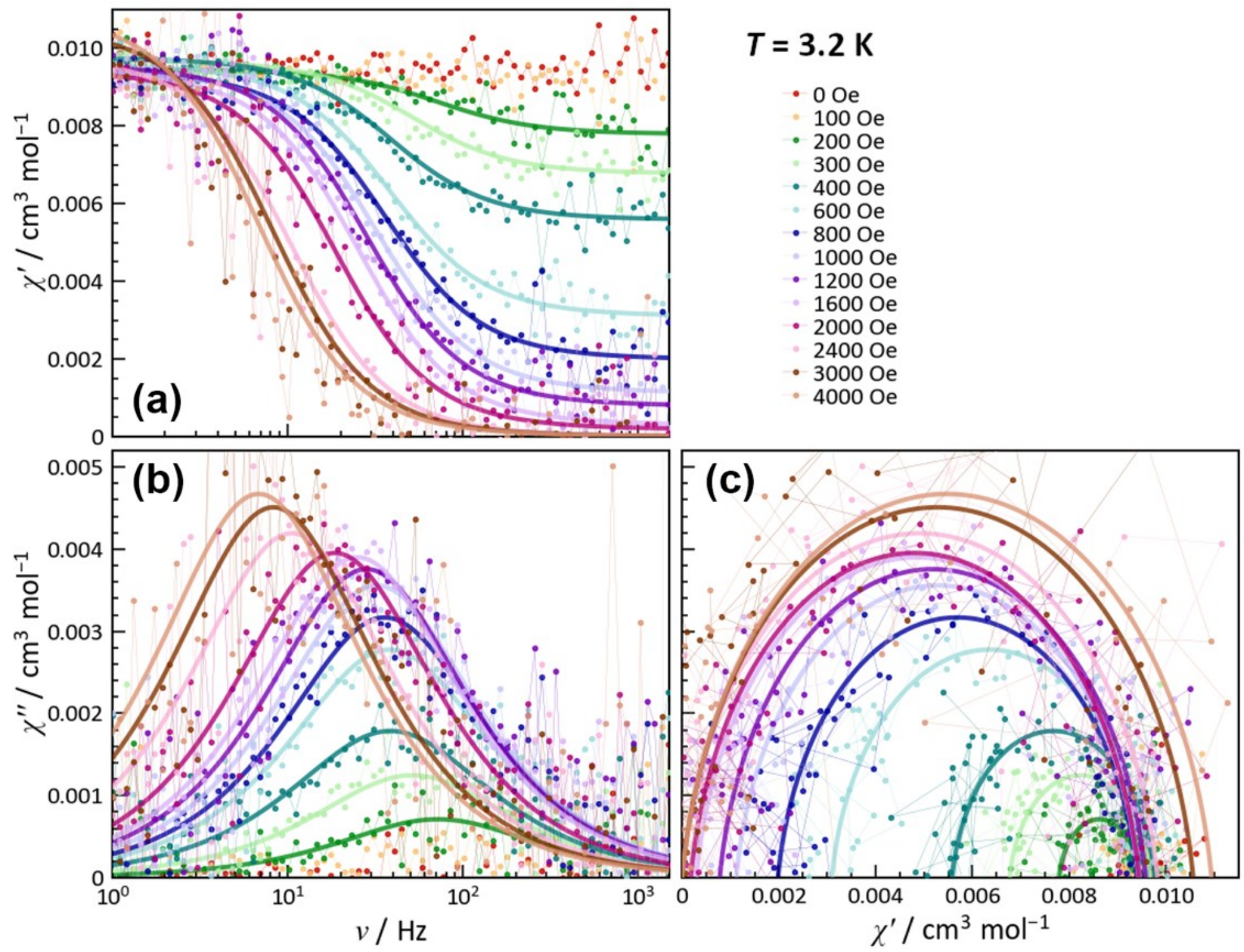

Figure S11. AC susceptibility data plotted as (a) χ' vs. ν, (b) χ'' vs. ν, and (c) χ'' vs. χ' (Cole-Cole plot) measured at 3.2 K under *H* between 0 and 4 kOe on the same sample as in **Figure S9**, and their best-fit curves using the generalized Debye model (solid traces).

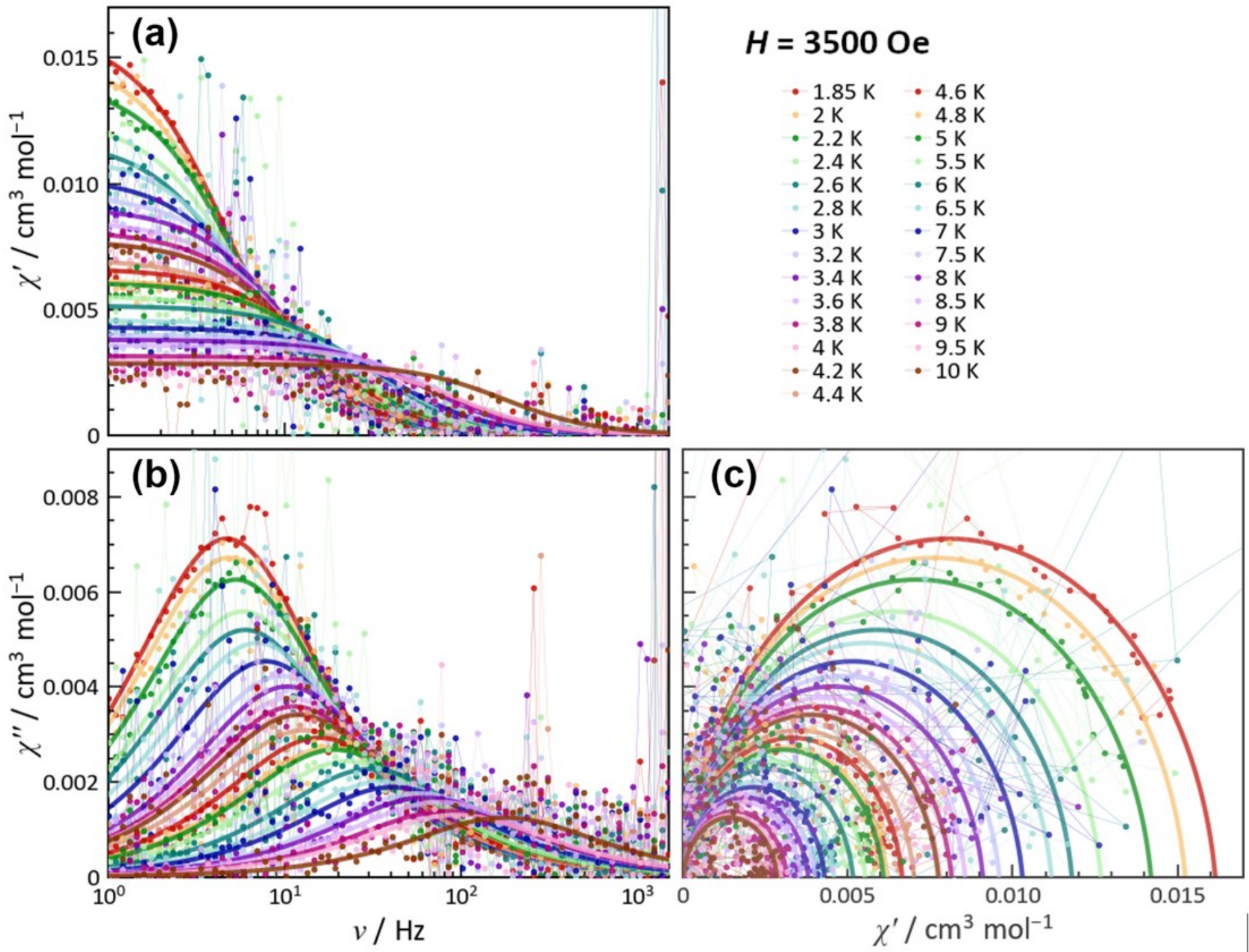

Figure S12. AC susceptibility data plotted as (a) χ' vs. ν, (b) χ'' vs. ν, and (c) χ'' vs. χ' (Cole-Cole plot) measured under *H* of 3.5 kOe between 1.85 and 10 K on the same sample as in **Figure S9**, and their best-fit curves using the generalized Debye model (solid traces).

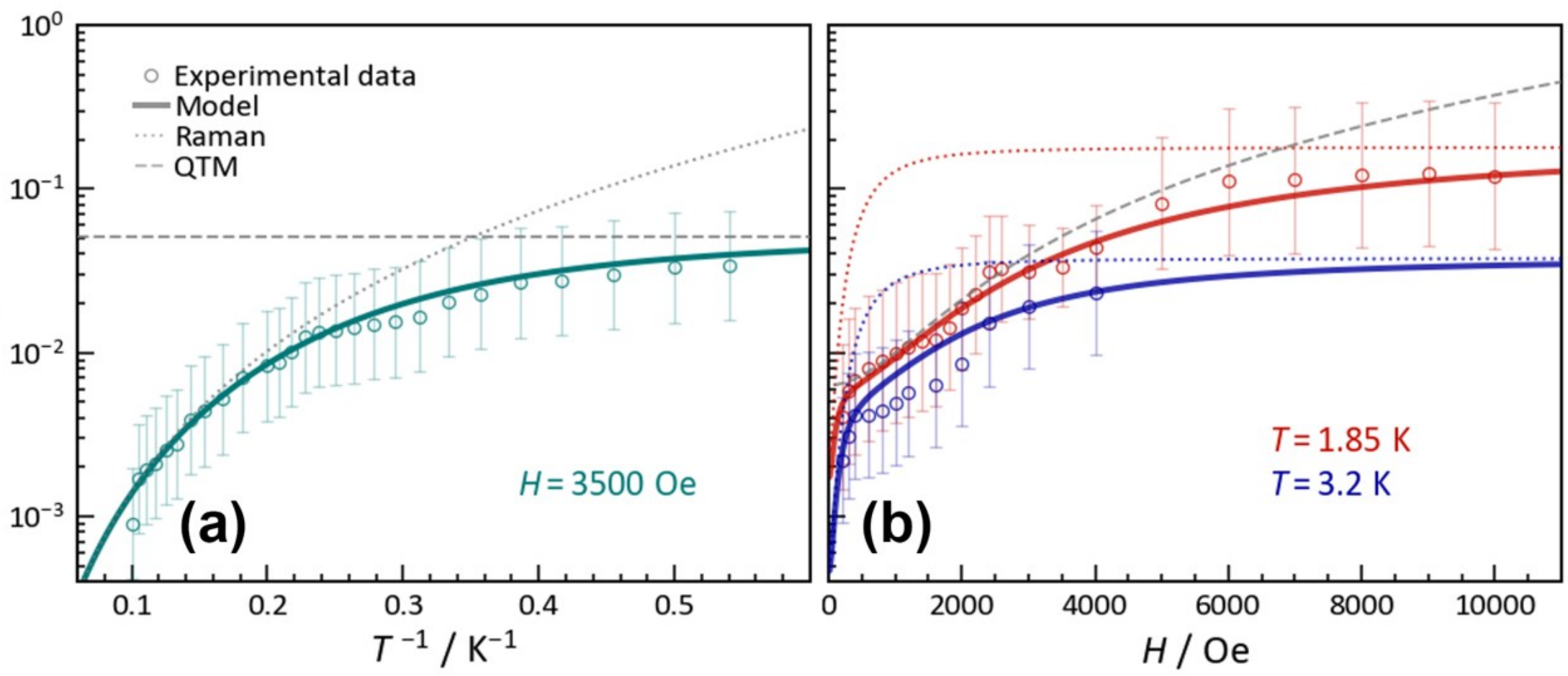

Figure S13. (a) Temperature dependence (at 3.5 kOe) and (b) field dependence (at 1.85 and 3.2 K) of the average relaxation time of the magnetization (τ) for **4**·2THF estimated from the data shown in **Figures S10**–**S12**. Open circles are the experimental data, and vertical solid bars denote ESDs of the distribution of times calculated from the α-parameters of the generalized Debye fits.[10] Solid lines are the result of the best global simulation (**eq S1**).

EPR and ENDOR details

Crystal sample orientation

The arrangement of molecules in the unit cell (**Figure S14a**) doesn't allow for probing them all in the parallel direction. Instead, the 4-fold axis was aligned with the static magnetic field, thereby affording the same orientation perpendicular to the V–Pt axis for all molecules. The optically isotropic crystal face normal to the *c* axis was identified using a polarizing microscope (see **Supplementary Video**).

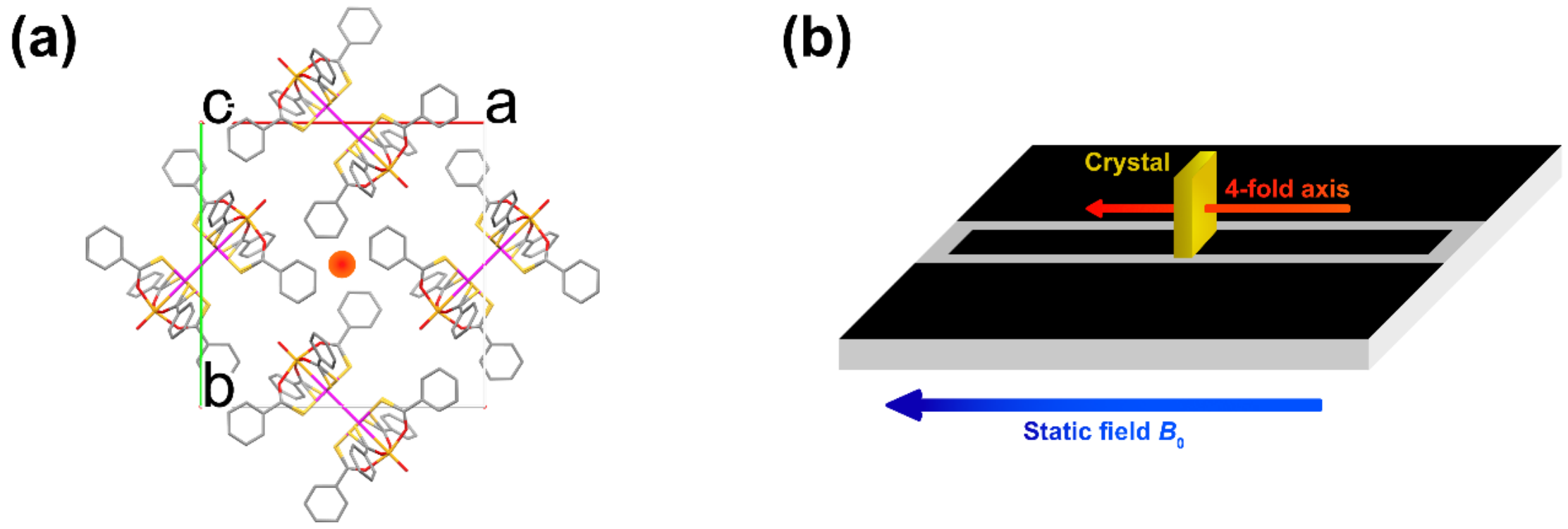

Figure S14. (a) Arrangement of the molecules around the 4-fold axis (red dot). (b) Orientation of the crystal on the superconducting YBCO resonator.

Supplementary note 3: EasySpin simulation of the CW-EPR

Simulations of the CW-EPR absorption spectrum (**Figure 3a**) and of the Zeeman diagram (**Figure 3b**) were performed using the EasySpin[11] toolbox with MATLAB software.

The initial values of the hyperfine tensors, including the values of all relevant magnetic interactions, were taken from Ref. [4]. In frozen solutions of **1**, components of the axial g-factor are g_z = 1.935 and $g_{x,y}$ = 1.991. The hyperfine interaction between the VO^{2+} electronic spin and ^{51}V nuclear spin has axial anisotropy, with components $^{V}A_z$ = –517.5 MHz and $^{V}A_{x,y}$ = –196.0 MHz. Finally, the superhyperfine interaction with the ^{195}Pt nuclei (natural abundance 34%) gives $^{Pt}A_z$ = –80 MHz and $^{Pt}A_{x,y}$ = –47 MHz. The positions of molecules relative to the applied fields were considered to match the structural arrangement in the **2**·2THF matrix ($I\bar{4}c2$ space group). Within the unit cell, molecules are orthogonal to the crystallographic *c* axis and are at 45° with respect to the *a* and *b* axes. The 4-fold symmetry axis, directed along the *c* axis, is oriented along the static field $\mathbf{B}_0$, resulting in the probing of all molecules in the direction perpendicular to the V–Pt axis (as shown in **Figure S14a**). Starting from these parameters and experimental conditions, a fit

is performed by fixing all values except the ones concerning the direction perpendicular to the molecular V–Pt axis. The fit reproduces the CW-EPR spectrum correctly, resulting in $g_{x,y}$ = 1.995 and $^{V}A_{x,y}$ = –204.3 MHz. These parameters were also used to extract all ENDOR resonance frequencies (energy differences between levels with $\Delta^{V}m_I = \pm 1$ and $\Delta m_S = 0$ for a fixed static magnetic field value) reported in **Table S3** and **Table S4**.

^{1}H ENDOR signal

To investigate the ENDOR signal of the ^{1}H nuclear spin, a sweep of the RF frequency was performed in the 8-14 MHz range using a Davies-ENDOR sequence. This measurement was repeated for four different values of B_0, corresponding to four different lines in the EPR spectrum shown in **Figure 3a** (I, IV, VII, VIII). The Larmor frequency of the proton (ν_H) increased with the static field value, according to:

$$\nu_H = \gamma B_0 \qquad \textbf{(S2)}$$

where γ is the gyromagnetic ratio with a value of 42.58 MHz/T. The resulting spectra, shown in **Figure S15**, are in excellent agreement with the expected behavior described by **eq S2**.

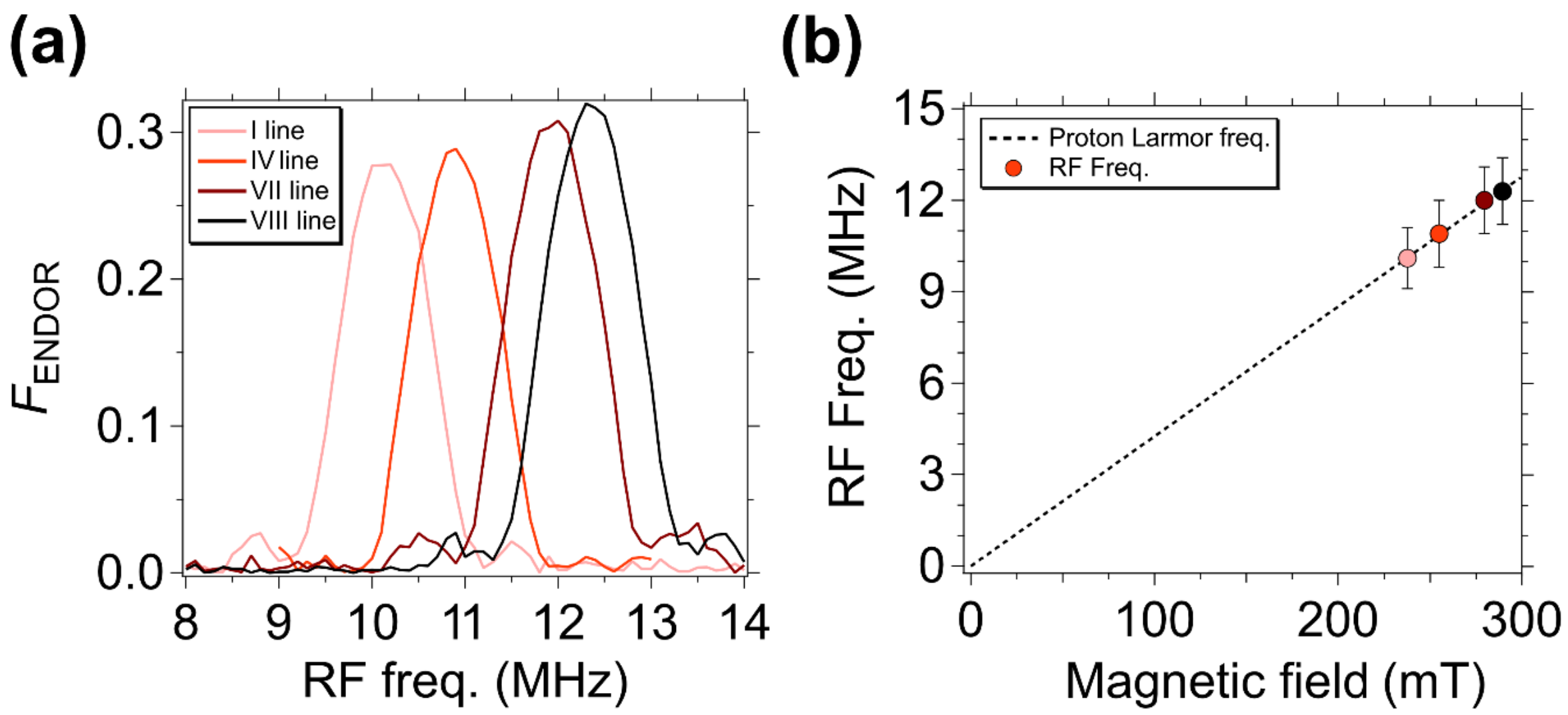

Figure S15. (a) ENDOR spectrum of ^{1}H nuclear spin taken at B_0 in resonance with different electronic transitions in the hyperfine manifold of **Figure 3a**. (b) Resonance frequency of the proton, corresponding to the maximum values of the peaks in (a). The errors are taken as the FWHM of each corresponding peak, while the expected Larmor frequency (**eq S2**) is plotted as a dashed line.

Additional ⁵¹V Mims-ENDOR spectra

The ENDOR signal of ^{51}V was measured for different electronic transitions in the hyperfine manifold, besides those reported in the main text. **Figure S16** shows the ENDOR signal measured for the I, IV, VII, and VIII electronic transitions (from the lowest to the largest magnetic field value) of the spectrum shown in **Figure 3a**.

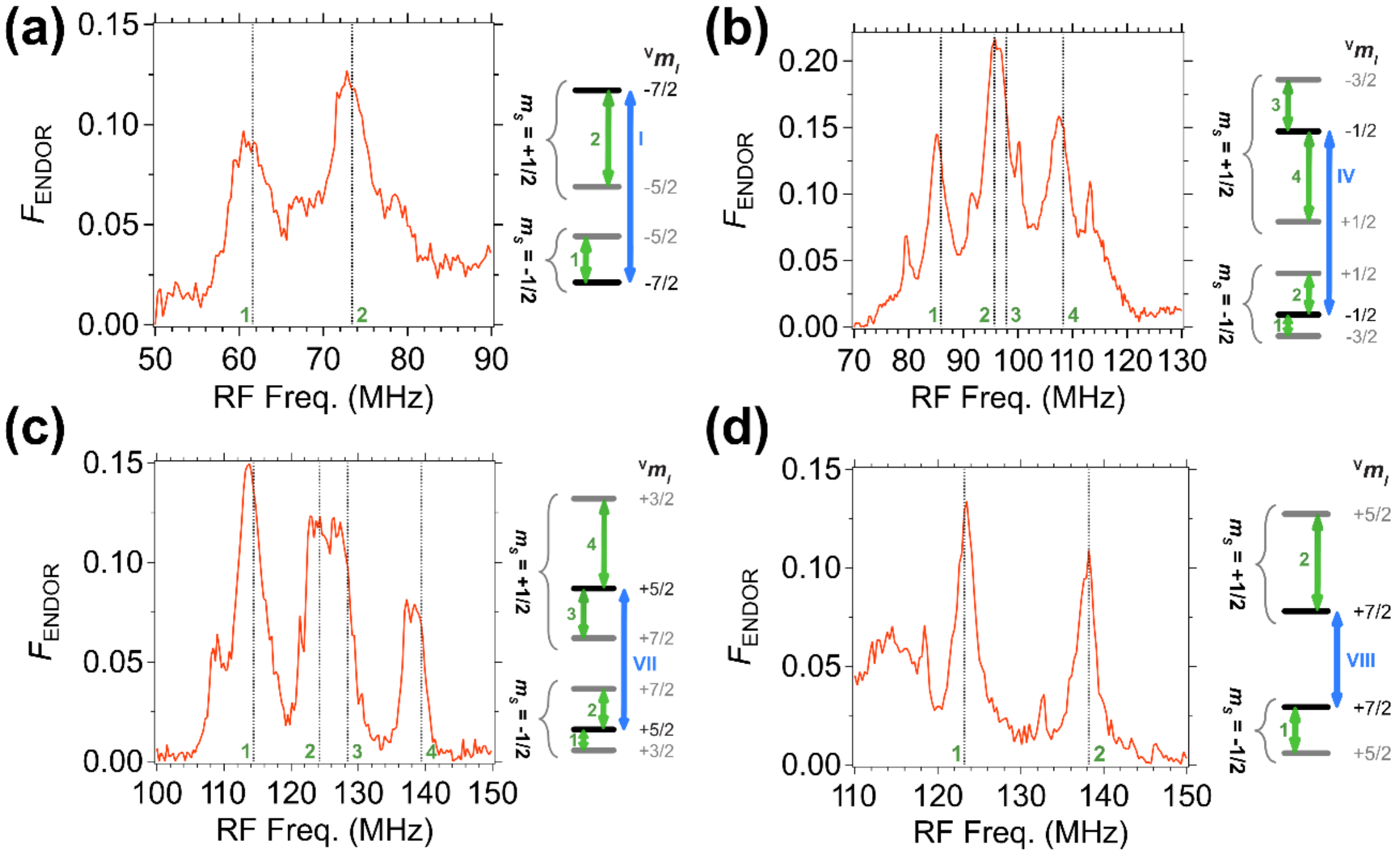

Figure S16. ENDOR signal of ^{51}V for different electronic transitions: I (B_0 = 237.7 mT) (a), IV (B_0 = 255.0 mT) (b), VII (B_0 = 279.5 mT) (c), and VIII (B_0 = 289.5 mT) (d), respectively. For all panels, vertical dashed lines show the nuclear resonance values calculated from simulations. The main ENDOR transitions addressed are shown in the sketch on the right of each panel and are indicated by green numbers.

Note that two main peaks are present for the I and VIII lines. In contrast, for the IV and VII lines, three main peaks are visible. These peaks correspond to the four available nuclear transitions involving the selected electronic levels, with the central one resulting from the overlap of two of them. All the transitions in **Figure S16** are listed in **Table S3**, together with the expected resonance frequencies obtained from EasySpin simulations using parameters given by the fit of the CW-ESR spectrum (**Figure 3a**).

It is known that Mims-ENDOR can give holes in the spectrum.[12] They manifest as periodic oscillations in the ENDOR efficiency with a period of $1/\tau \approx 0.7$ MHz (being τ = 1500 ns the interpulse delay between the first two $\pi/2$ pulses, see **Figure 4a**). This value is too small to

explain the energy difference between the main peaks observed, which can be safely attributed to ^{51}V.

Table S3. Summary of all ENDOR transitions reported in **Figure S16**. The experimentally measured frequencies (**Figure 4** and **Figure S16**) are taken at each peak maximum, while the associated uncertainties are estimated as the FWHM of the corresponding peak. For overlapping peaks, the uncertainties are taken as the FWHM of the unresolved peak.

e^- transition	nuclear transition $(m_S, {}^{V}m_I) \rightarrow (m_S, {}^{V}m_I)$	Calculated freq. (MHz)	Measured freq. (MHz)
**I** (B_0 = 237.7 mT)	**1** (−1/2, −5/2) → (−1/2, −7/2)	61.6	61.1 ± 6.0
	**2** (+1/2, −7/2) → (+1/2, −5/2)	73.4	72.8 ± 7.2
**IV** (B_0 = 255.0 mT)	**1** (−1/2, −3/2) → (−1/2, −1/2)	85.9	85.0 ± 4.8
	**2** (−1/2, −1/2) → (−1/2, +1/2)	95.7	95.8 ± 7.8
	**3** (+1/2, −1/2) → (+1/2, −3/2)	97.9	96.7 ± 7.8
	**4** (+1/2, +1/2) → (+1/2, −1/2)	108.3	107.8 ± 3.5
**VII** (B_0 = 279.5 mT)	**1** (−1/2, +3/2) → (−1/2, +5/2)	114.4	113.8 ± 4.8
	**2** (−1/2, +5/2) → (−1/2, +7/2)	124.2	123.7 ± 7.6
	**3** (+1/2, +7/2) → (+1/2, +5/2)	128.4	127.0 ± 7.6
	**4** (+1/2, +5/2) → (+1/2, +3/2)	139.4	138.1 ± 3.9
**VIII** (B_0 = 289.5 mT)	**1** (−1/2, +5/2) → (−1/2, +7/2)	123.2	123.5 ± 3.3
	**2** (+1/2, +7/2) → (+1/2, +5/2)	138.2	138.2 ± 3.0

Supplementary note 4: ^{195}Pt contribution to Mims-ENDOR spectra

A fraction of the ^{195}Pt isotope (natural abundance 34%) causes a superhyperfine coupling in molecules containing it with tensor parameters ${}^{Pt}A_z$ = −80 MHz and ${}^{Pt}A_{x,y}$ = −47 MHz.[4] This interaction splits each of the 16 hyperfine levels into a further 32 levels in total. However, the transitions listed in **Table S4** alongside the expected resonance frequencies obtained from EasySpin simulations are too close in energy to the transitions of the main ENDOR peaks (**Figure S17**). Therefore, the ^{195}Pt contribution to the ENDOR spectra was not directly observable.

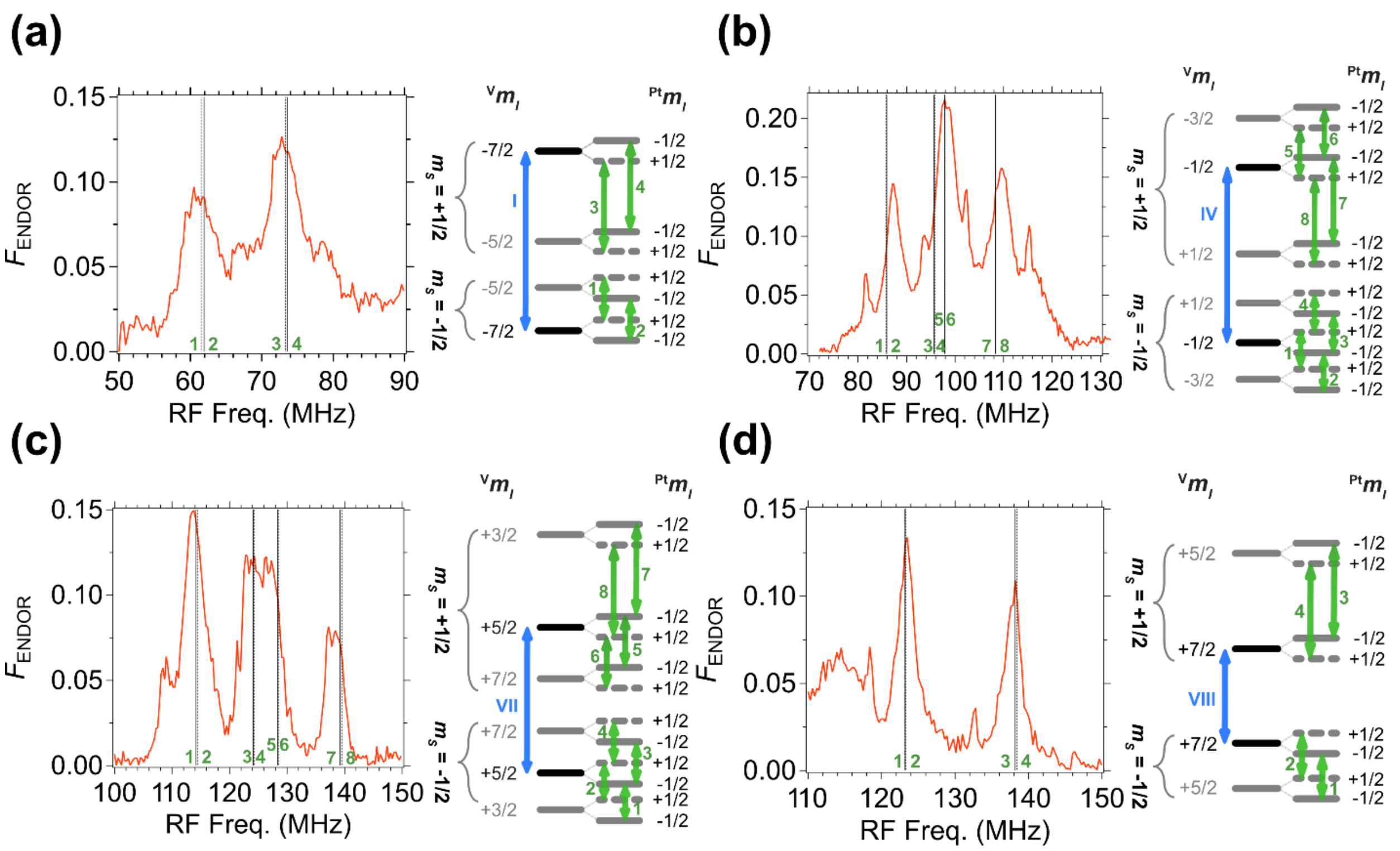

Figure S17. ENDOR signal of ^{51}V for different electronic transitions of **Figure 3a**. The electronic transitions correspond to I (B_0 = 237.7 mT) (a), IV (B_0 = 255.0 mT) (b), VII (B_0 = 279.5 mT) (c), and VIII (B_0 = 289.5 mT) (d) lines, respectively. The sketches in all panels show the superhyperfine interaction leading to further splitting into $^{Pt}m_I$ = +1/2 ($^{Pt}m_I$ = −1/2) states represented by dashed (solid) lines. Vertical dashed (solid) lines show the nuclear resonance frequencies calculated from simulations.

Table S4. Summary of all ENDOR transitions reported in **Figure S17** for $\Delta^{V}m_I = \pm 1$, $\Delta^{Pt}m_I = 0$ in the presence of ^{195}Pt superhyperfine interaction. The ENDOR resonances are calculated starting from the EasySpin Zeeman diagram (**Figure 3b**).

**e⁻ transition**	**nuclear transition** (m_s, $^{V}m_I$, $^{Pt}m_I$) → (m_s, $^{V}m_I$, $^{Pt}m_I$)	Calculated freq. (MHz)
**I** (B_0 = 237.7 mT)	**1** (−1/2, −7/2, +1/2) → (−1/2, −5/2, +1/2)	61.5
	**2** (−1/2, −7/2, −1/2) → (−1/2, −5/2, −1/2)	61.9
	**3** (+1/2, −5/2, +1/2) → (+1/2, −7/2, +1/2)	73.3
	**4** (+1/2, −5/2, −1/2) → (+1/2, −7/2, −1/2)	73.6
**IV** (B_0 = 255.0 mT)	**1** (−1/2, −3/2, +1/2) → (−1/2, −1/2, +1/2)	85.8
	**2** (−1/2, −3/2, −1/2) → (−1/2, −1/2, −1/2)	85.9
	**3** (−1/2, −1/2, −1/2) → (−1/2, +1/2, −1/2)	95.7
	**4** (−1/2, −1/2, +1/2) → (−1/2, +1/2, +1/2)	95.8
	**5** (+1/2, −1/2, +1/2) → (+1/2, −3/2, +1/2)	97.9
	**6** (+1/2, −1/2, −1/2) → (+1/2, −3/2, −1/2)	97.9
	**7** (+1/2, +1/2, −1/2) → (+1/2, −1/2, −1/2)	108.3
	**8** (+1/2, +1/2, +1/2) → (+1/2, −1/2, +1/2)	108.4

e⁻ transition	nuclear transition (m_s, ${}^{V}m_I$, ${}^{Pt}m_I$) → (m_s, ${}^{V}m_I$, ${}^{Pt}m_I$)	Calculated freq. (MHz)
**VII** (B_0 = 279.5 mT)	**1** (−1/2, +3/2, −1/2) → (−1/2, +5/2, −1/2)	114.2
	**2** (−1/2, +3/2, +1/2) → (−1/2, +5/2, +1/2)	114.4
	**3** (−1/2, +5/2, −1/2) → (−1/2, +7/2, −1/2)	124.1
	**4** (−1/2, +5/2, +1/2) → (−1/2, +7/2, +1/2)	124.3
	**5** (+1/2, +5/2, −1/2) → (+1/2, +3/2, −1/2)	128.3
	**6** (+1/2, +5/2, +1/2) → (+1/2, +3/2, +1/2)	128.5
	**7** (+1/2, +7/2, −1/2) → (+1/2, +5/2, −1/2)	139.2
	**8** (+1/2, +7/2, +1/2) → (+1/2, +5/2, +1/2)	139.5
**VIII** (B_0 = 289.5 mT)	**1** (−1/2, +5/2, −1/2) → (−1/2, +7/2, −1/2)	123.2
	**2** (−1/2, +5/2, +1/2) → (−1/2, +7/2, +1/2)	123.3
	**3** (+1/2, +7/2, −1/2) → (+1/2, +5/2, −1/2)	138.1
	**4** (+1/2, +7/2, +1/2) → (+1/2, +5/2, +1/2)	138.4

Comparison between Davies-ENDOR and Mims-ENDOR

The Davies-ENDOR sequence was also tested. While a decrease in echo amplitude was observed with Mims (**Figure S18a**), an increase was found with Davies (**Figure S18b**), as expected.[12] Plotting each spectrum as ENDOR efficiency (F_{ENDOR} , **eq 2**) vs RF frequency allows for a direct comparison of the position of the peaks (**Figure S18c**). Both sequences give comparable spectroscopic features under our experimental conditions. However, the peaks are better resolved when the Mims sequence is applied, and for this reason, this protocol is chosen as the main one for ENDOR measurements.

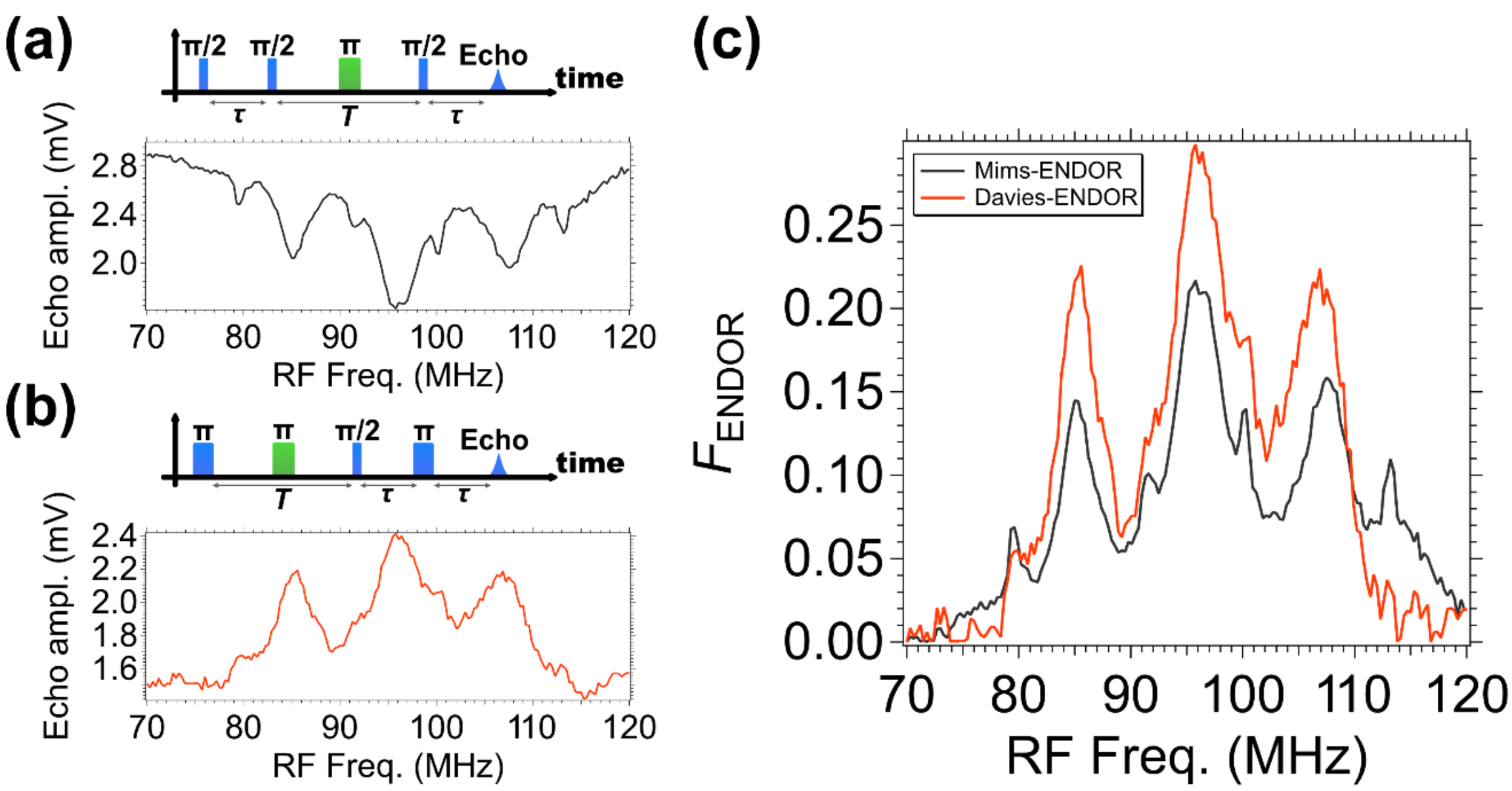

Figure S18. ENDOR spectrum of ${}^{51}V$ taken at the static field B_0 = 255.0 mT at 3K, corresponding to the IV electronic transition in the hyperfine manifold with a Mims-ENDOR sequence (a) and with a Davies-ENDOR sequence (b). (c) Spectra (a) and (b), converted to F_{ENDOR} for comparison.

Test of storage block efficiency

To test the efficiency of the storage block (MW π/2; MW π; RF π; MW π, see **Figure 5b**), the spectra obtained with a Mims-ENDOR sequence (**Figure S17d**) and an SR sequence with only the first RF π pulse applied (**Figure S19**) were compared. The VIII electronic transition (B_0 = 289.0 mT) was used due to its simpler pattern with no overlapping peaks (**Figure S16d**). The ENDOR efficiency, F_{ENDOR} (**eq 2**), shows that both the positions and the shapes of the peaks match well (**Figure S19c**), indicating that the storage block acts similarly to a standard ENDOR sequence, i.e., it allows polarization transfer between states involving the selected electronic transition.

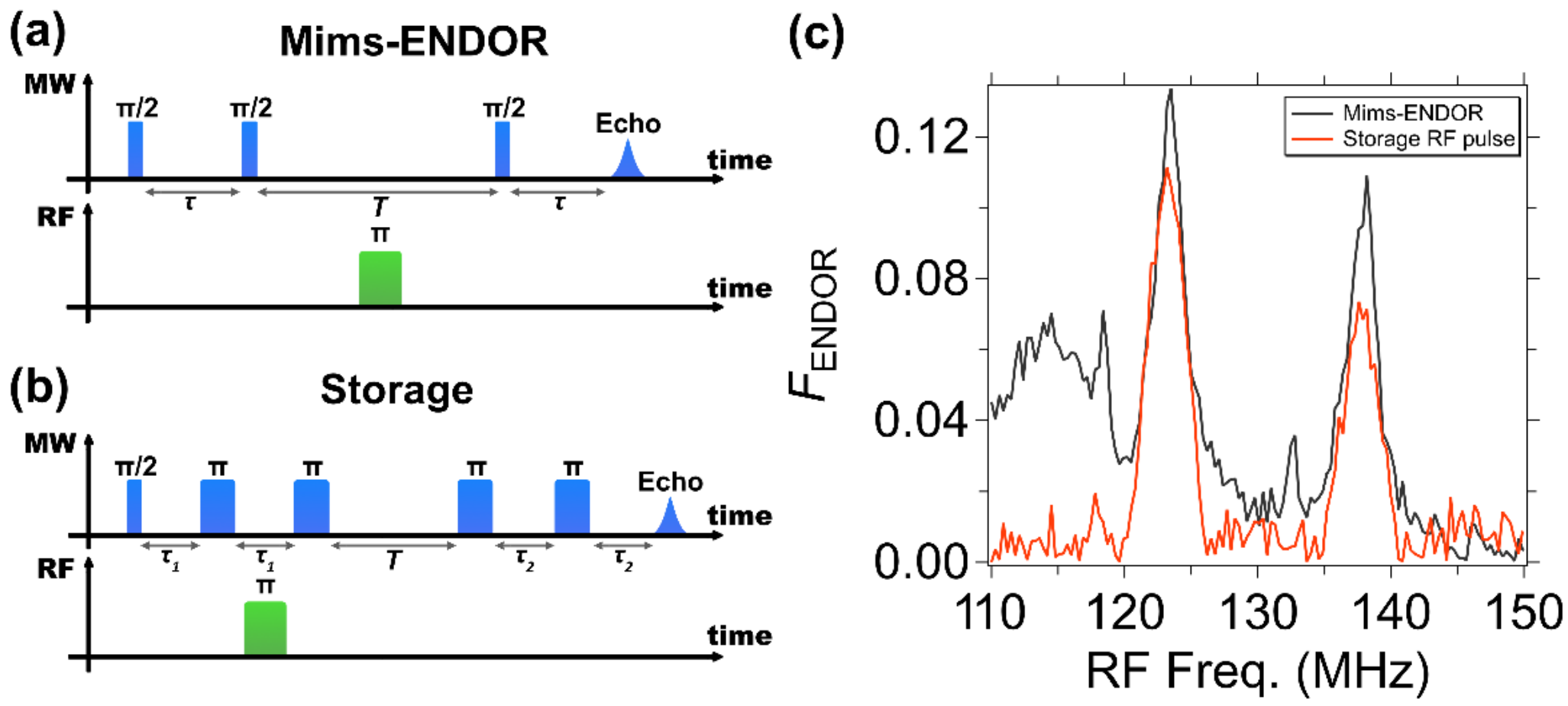

Figure S19. (a) Mims-ENDOR sequence. (b) SR sequence with only the first RF π pulse ON. (c) Comparison between the amplitude of the echo signals resulting from sequences (a) and (b) expressed as F_{ENDOR}.

Tests of the full SR protocol

To assess the effectiveness of the SR protocol, quadrature measurements were performed on the electron spin echo appearing at the end of the protocol. This allows for the extraction of the I and Q quadrature signals of the echo, corresponding to the S_x and S_y spin components. The signal obtained with a Hahn echo sequence and τ ~ 1.8 μs was used as a reference for the correct behavior of the signal quadrature, since for this short time, the phase imposed by the first MW π/2 pulse can be maintained by the electronic spin (**Figure S20a** and **d**). Conversely, using τ ~ 9 μs, largely exceeding the electronic T_m, the echo signal was lost, as evidenced by the quadrature signals in **Figure S20b** and **e**. Finally, in an SR sequence with all RF pulses turned off, the resulting quadrature signals did not match the encoded phase (**Figure S20c** and **f**). Overall, only when a full SR sequence was applied in resonance with one of the nuclear transitions was coherence safely retrieved after a total free precession time much longer than T_m (**Figure 6c-f** and **Figure S21b-d**).

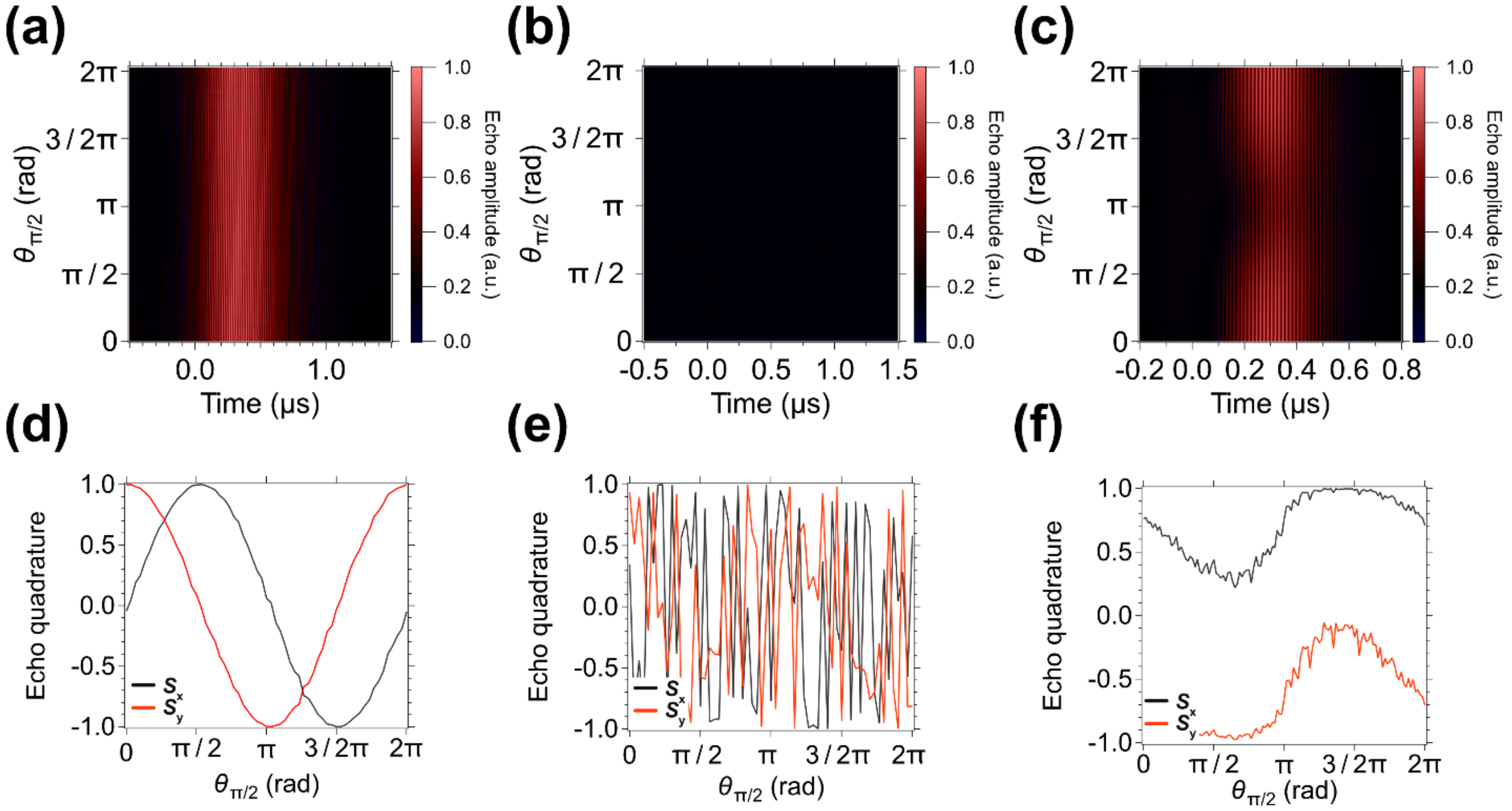

Figure S20. Map of the echo amplitude as a function of time and phase of the first MW π/2 pulse (top) and corresponding echo quadrature signals extracted as a function of the phase of the first π/2 pulse (bottom). Reference traces obtained with a Hahn echo sequence with an interpulse delay τ = 1.8 µs (a,d). Traces obtained with a Hahn echo sequence with an interpulse delay $\tau \sim$ 9 µs > T_m, (b,e). Traces obtained from an SR protocol with all RF pulses OFF (c,f).

To further examine the use of nuclear spins as a memory, the SR sequence was applied with increasing values of the central delay T (10, 30, and 50 µs), all exceeding T_m. The extracted echo components (**Figure S21**) exhibit behavior comparable to that obtained from the reference signal in **Figure 6a**, with the signal becoming noisier for longer T values.

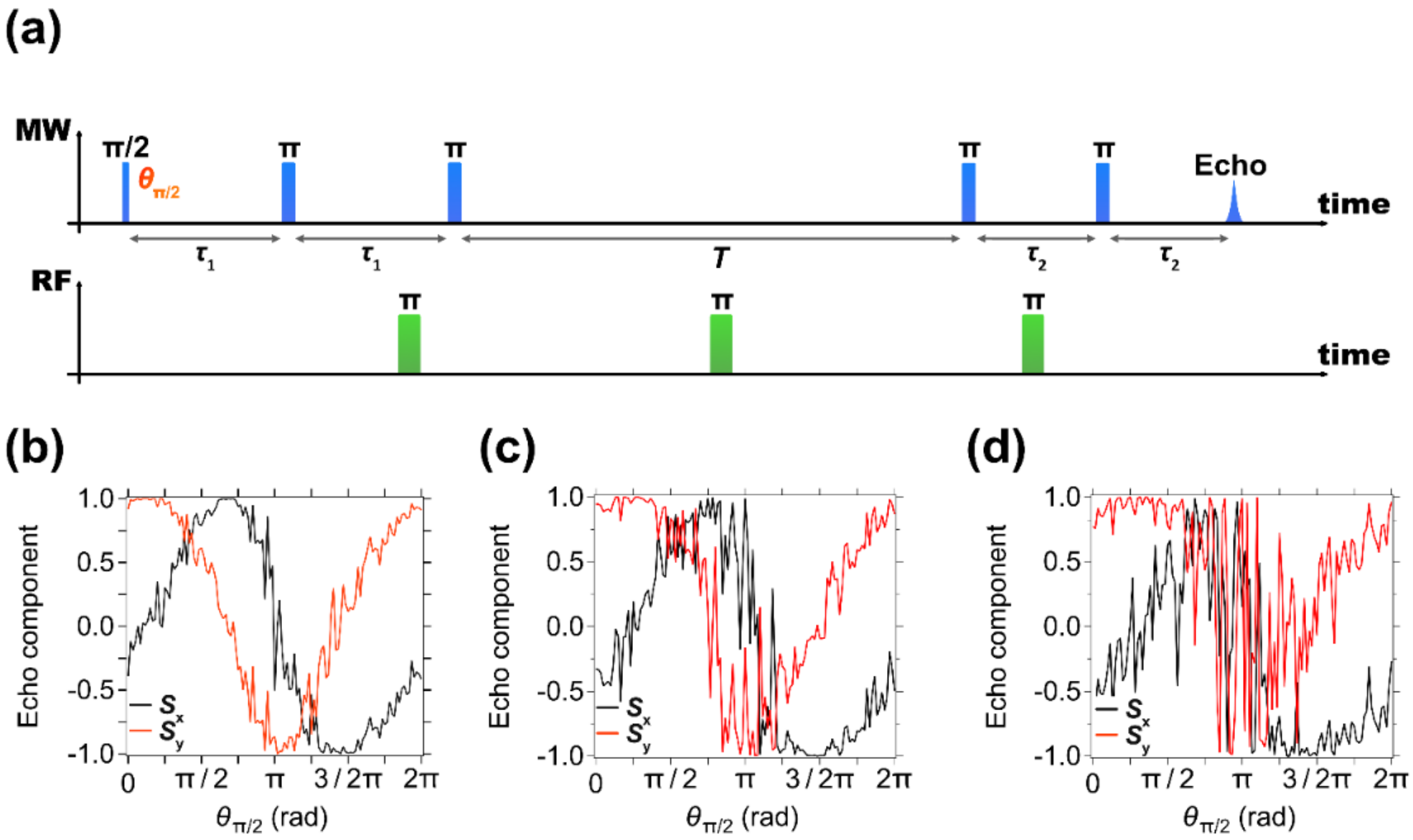

Figure S21. (a) SR sequence applied for increasing values of the central delay T (10, 30, and 50 µs). Resulting echo component extracted from the obtained electron spin echo (b, c, d).

Supplementary note 5: Estimation of in-plane fidelity of the SR protocol

To quantify the performance of the protocol and the changes at different sequence durations, a fidelity-like parameter, referred to hereafter as *in-plane fidelity* (f), has been defined as the deviation of the phase of the output echo from its value encoded as input:

$$f = (360 - |\varphi - \varphi_{\text{init}}|)/360 \quad \textbf{(S3)}$$

where φ is the phase of the echo, defined as $\varphi = \arctan(S_y/S_x)$, and φ_{init} is the phase value given by the initialization performed by the first MW pulse. This parameter takes into account only the precession plane of the spins (S_x and S_y), and not the reconstruction of the full output state. f is normalized over the range of values that the phase can assume (from 0 to 360 deg). As a result, this parameter is 1 when the experimental value exactly matches the expected one. f is extracted for all points of the measurements reported in **Figure 6** and **Figure S21**. The resulting f curve is averaged over all values of the 1st MW pulse phase to extract an average value across the full 360-degree initialization span. The results, summarized in **Figure S22** as a function of the central delay T used in the SR protocols, show that f decreases from ~0.97 at 4 µs to ~0.87 at 50 µs.

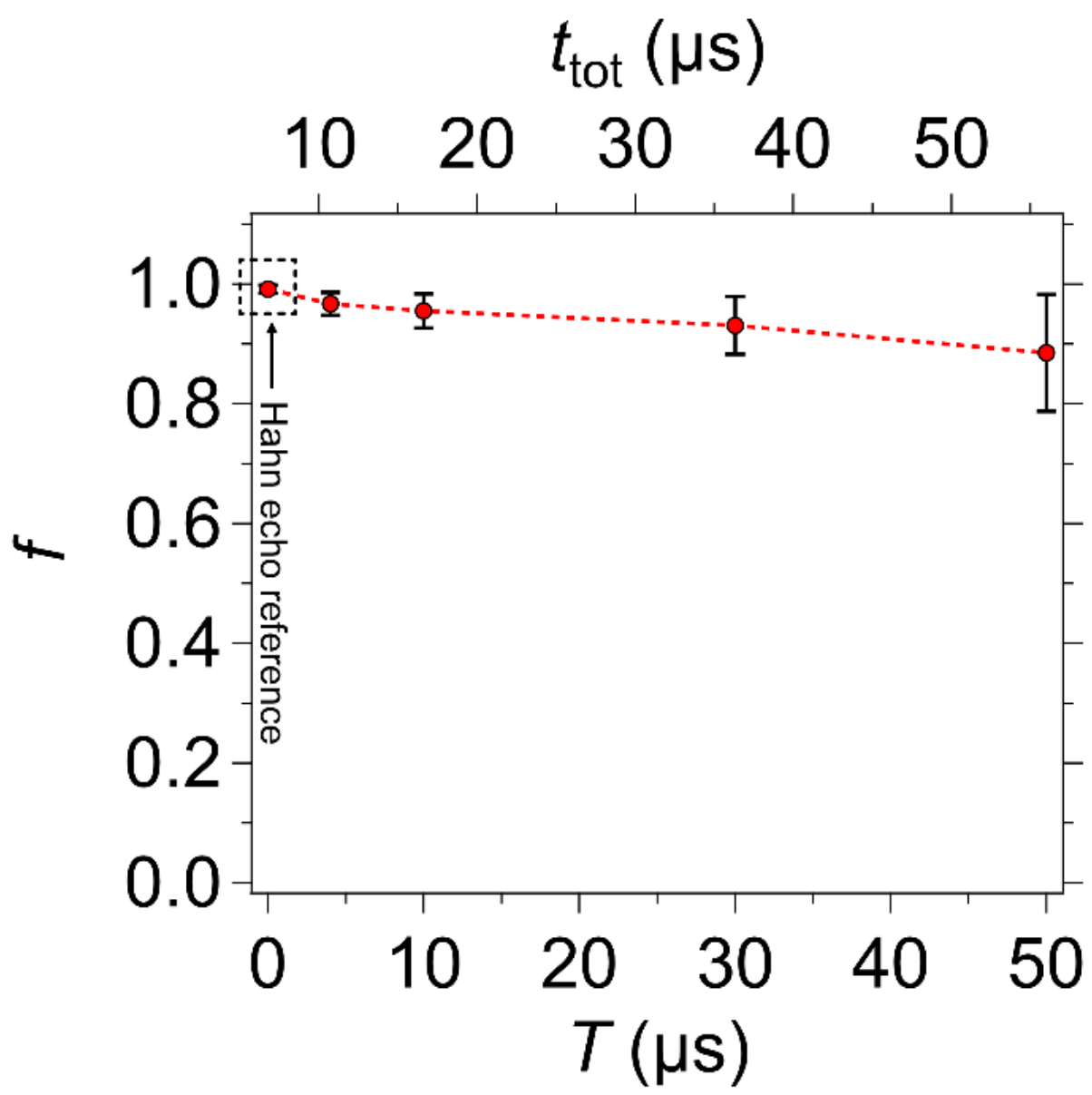

Figure S22. *In-plane fidelity* for the measurements reported in **Figure 6** and **Figure S21**. The values are averages taken over all values of the 1st MW pulse phase initialization (from 0 to 360 deg) for each delay T used; the error bars correspond to the standard deviation of each average. The top scale shows the total precession time (t_{tot}).

Calibration of the duration of the RF pulses

The duration of the RF pulses used in all ENDOR and SR protocols was calibrated using the sequence shown in **Figure S23a**. In this modified Mims-ENDOR sequence, the RF pulse duration is swept step-by-step at a fixed amplitude. The RF pulse drives nuclear Rabi oscillations, resulting in the maximum effect (i.e., maximal ENDOR efficiency, F_{ENDOR}) for a duration T_{RF} corresponding to an effective RF π pulse. For the electronic transition corresponding to $m_S = -1/2 \rightarrow m_S = +1/2$ (B_0 = 255.0 mT) at a fixed RF frequency of 85 MHz, in resonance with the ${}^{V}m_I = -3/2 \rightarrow {}^{V}m_I = -1/2$ nuclear transition (**Figure S23b** and **Table S3**), the maximum ENDOR efficiency was found for T_{RF} = 250 ns (**Figure S23c**). The correct RF duration can be different for distinct nuclear transitions, so this calibration process can be repeated each time to ensure an optimal value of T_{RF}.

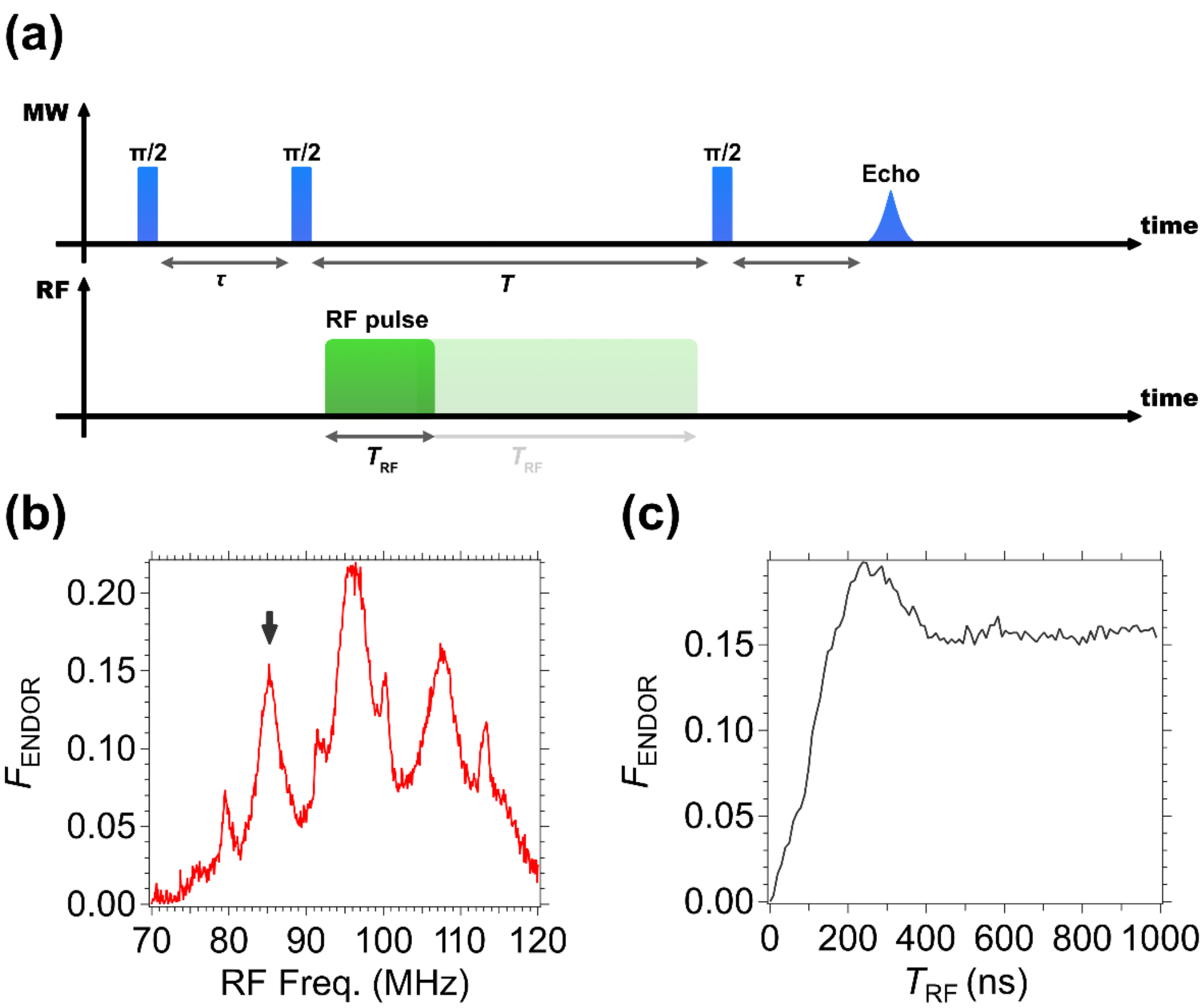

Figure S23. (a) Mims-ENDOR sequence used for the calibration of the duration of the RF pulse. (b) ENDOR spectrum taken for the electronic transition $m_S = -1/2 \rightarrow m_S = +1/2$ (B_0 = 255.0 mT), with target ENDOR transition ${}^{V}m_I = -3/2 \rightarrow {}^{V}m_I = -1/2$ (black arrow, corresponding to 85.80 MHz, see **Table S3**). (c) ENDOR signal obtained with the sequence in (a) as a function of T_{RF}.

References

(1) Spek, A. L. PLATON SQUEEZE: A Tool for the Calculation of the Disordered Solvent Contribution to the Calculated Structure Factors. *Acta Crystallogr. Sect. C: Struct. Chem.* **2015**, *71*, 9–18. https://doi.org/10.1107/S2053229614024929.

(2) Spek, A. L. Single-Crystal Structure Validation with the Program PLATON. *J. Appl. Crystallogr.* **2003**, *36*, 7–13. https://doi.org/10.1107/S0021889802022112.

(3) Mironova, O.; Bellini, G.; Nicolini, A.; Cornia, A. Multiple Dimerization Modes in Thiocarboxylate Paddlewheel Complexes: A Comprehensive View of Energy Landscapes from DFT Calculations and Statistics. *Chem. Eur. J.* **2025**, e03004. https://doi.org/10.1002/chem.202503004.

(4) Imperato, M.; Nicolini, A.; Borsari, M.; Briganti, M.; Chiesa, M.; Liao, Y.-K.; Ranieri, A.; Raza, A.; Salvadori, E.; Sorace, L.; Cornia, A. Quantum Spin Coherence and Electron Spin Distribution Channels in Vanadyl-Containing Lantern Complexes. *Inorg. Chem. Front.* **2024**, *11* (1), 186–195. https://doi.org/10.1039/d3qi01806g.

(5) Neufeld, R.; Stalke, D. Accurate Molecular Weight Determination of Small Molecules via DOSY-NMR by Using External Calibration Curves with Normalized Diffusion Coefficients. *Chem. Sci.* **2015**, *6* (6), 3354–3364. https://doi.org/10.1039/c5sc00670h.

(6) Bachmann, S.; Neufeld, R.; Dzemski, M.; Stalke, D. New External Calibration Curves (ECCs) for the Estimation of Molecular Weights in Various Common NMR Solvents. *Chem. Eur. J.* **2016**, *22* (25), 8462–8465. https://doi.org/10.1002/chem.201601145.

(7) Crockett, M. P.; Zhang, H.; Thomas, C. M.; Byers, J. A. Adding Diffusion Ordered NMR Spectroscopy (DOSY) to the Arsenal for Characterizing Paramagnetic Complexes. *Chem. Commun.* **2019**, *55* (96), 14426–14429. https://doi.org/10.1039/c9cc08229h.

(8) Mironova, O.; Bellini, G.; Nicolini, A.; Imperato, M.; Ranieri, A.; Borsari, M.; Briganti, M.; Clérac, R.; Rouzières, M.; Salvadori, E.; Pagliero, M. C.; Chiesa, M.; Cornia, A. Oxovanadium (IV) Thiocarboxylate Paddlewheels Containing Ancillary Group 10 Metals: A Comparative Study on Pd and Pt Derivatives. *Inorg. Chem.* **2026**, *65*, 638–651. https://doi.org/10.1021/acs.inorgchem.5c04835.

(9) Rouzières, M. PyMagSuite_V1.5.3. *Zenodo* **2020**. https://doi.org/doi.org/10.5281/zenodo.4030310.

(10) Blackmore, W. J. A.; Gransbury, G. K.; Evans, P.; Kragskow, J. G. C.; Mills, D. P.; Chilton, N. F. Characterisation of Magnetic Relaxation on Extremely Long Timescales. *Phys. Chem.*

Chem. Phys. **2023**, *25*, 16735–16744. https://doi.org/10.1039/d3cp01278f.

(11) Stoll, S.; Schweiger, A. EasySpin, a Comprehensive Software Package for Spectral Simulation and Analysis in EPR. *J. Magn. Reson.* **2006**, *178*, 42–55. https://doi.org/10.1016/j.jmr.2005.08.013.

(12) Goldfarb, D.; Stoll, S. EPR Spectroscopy: Fundamentals and Methods; *John Wiley & Sons*, **2018**.
